# Chemokine receptor 7 mediates miRNA‐182 to regulate cerebral ischemia/reperfusion injury in rats

**DOI:** 10.1111/cns.14056

**Published:** 2022-12-15

**Authors:** Qi Wang, Sifan Xu, Bin Wang, Yu Qin, Yachen Ji, Qian Yang, Yang Xu, Zhiming Zhou

**Affiliations:** ^1^ Department of Neurology, The First Affiliated Hospital of Wannan Medical College Yijishan Hospital Wuhu China; ^2^ Key Laboratory of Noncoding RNA Transformation Research of Anhui Higher Education Institutes Wannan Medical College Wuhu China; ^3^ Department of Anesthesiology, The First Affiliated Hospital of Wannan Medical College Yijishan Hospital Wuhu China

**Keywords:** cerebral ischemia/reperfusion injury, chemokine receptor 7, hippo pathway, microRNA

## Abstract

**Aims:**

Chemokine receptor 7 (CXCR7) exerts protective effects on the brain. MicroRNAs (miRNAs) are involved in cerebral ischemia/reperfusion (I/R) injury, but their involvement in CXCR7‐mediated brain protection is unknown. In this study, we investigated the role of miRNAs in CXCR7‐mediated brain protection.

**Methods:**

CXCR7 levels in peripheral blood samples from patients with acute ischemic stroke (AIS) and ischemic penumbra area brain tissues from middle cerebral artery occlusion (MCAO) rats after recanalization were measured. An miRNA microarray analysis was performed to examine the expression of miRNAs caused by CXCR7 knockdown in ischemic penumbra area brain tissue in middle cerebral artery occlusion–reperfusion rats and to predict corresponding downstream target genes. Kyoto Encyclopedia of Genes and Genomes (KEGG) pathway enrichment analysis revealed the most enriched pathways. A dual‐luciferase reporter assay confirmed the direct regulation of miR‐182 on the target gene TCF7L2. The correlation between TCF7L2 and CXCR7/miR‐182 was verified using rescue assays.

**Results:**

CXCR7 expression was upregulated in MCAO rats and mechanical thrombectomy patients with AIS compared to that in controls. The motor and sensory functions of MCAO rats with CXCR7 knockdown further decreased, and the infarct volume and cerebral edema increased. miRNA microarray data showed that seven miRNAs were differentially expressed after shRNA‐CXCR7 treatment. The dual‐luciferase reporter assay confirmed that miR‐182 directly targeted the TCF7L2 gene. Rescue assays confirmed that TCF7L2 is downstream of CXCR7/miR‐182. KEGG pathway analysis showed that the Hippo pathway may be a key pathway in CXCR7 upregulation and plays a role in protecting the brain after interventional surgery. Animal experiments have shown that CXCR7‐mediated cerebral I/R injury promotes the phosphorylation of key molecules YAP and TAZ in the Hippo pathway.

**Conclusion:**

CXCR7 protects against cerebral I/R injury, possibly via the miR‐182/TCF7L2/Hippo pathway. These results indicate that CXCR7 affects cerebral ischemia–reperfusion injury through miRNA regulation and downstream pathways.

## Graphical abstract

Ischemic stroke (IS) is a major cause of disability and mortality worldwide. CXCR7 has shown remarkable expression changes on cerebral ischemia–reperfusion (I/R) injury. Knockdown CXCR7 could significantly worsen neurological outcome, infarct size, and cerebral edema. The CXCR7/miR‐182/TCF7L2/Hippo regulatory axis may be a novel strategy to deal with cerebral I/R injury.

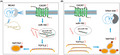

## 1 INTRODUCTION

Acute ischemic stroke (AIS) is the main type of stroke, accounting for approximately 80% of all cases.^1^, ^2^ Currently, perfusion recovery therapy is the most effective strategy for reducing infarct volume and improving clinical efficacy.^3^ However, brain injury typically continues to progress after reperfusion, leading to ischemia/reperfusion (I/R) injury,^4^ and is induced by inflammatory response, excitotoxicity, and reactive oxygen species, resulting in irreversible damage to important organelles and DNA.^5^, ^6^ Although various studies have attempted to explain the pathogenesis of I/R injury, clinical treatment remains a challenge. Therefore, exploring the mechanisms of I/R injury is critical, as the results may provide a theoretical basis for finding new therapeutic targets for I/R injury.

Increasing evidence has shown that chemokines are involved in I/R injury,^7^ and that sustained overproduction of chemokine signaling in a cerebral I/R injury region drives chemokine signaling, leading to the accumulation and homing of endothelial cells to the injury site.^8^, ^9^, ^10^ Stromal‐derived factor‐1 (SDF‐1), a member of the chemotactic cytokine superfamily, is widely expressed in the central nervous system. The specific receptor cysteine‐X‐cysteine chemokine receptor 4 (CXCR4) has been shown to play a role in cardiovascular and cerebrovascular diseases.^11^ Balabanian identified CXCR7 as the second receptor for CXCL12^12^ and CXCR7 has been shown to have a higher affinity for ligands than the classical receptor CXCR4.^13^ Furthermore, CXCR7 regulates atherosclerotic lesion formation^14^ and loss of CXCR7 impairs vascular hemostasis.^15^ CXCR7 is considered to be the main receptor of SDF‐1 after stroke^16^, ^17^ and is a useful tool in biological research,^18^ especially as a therapeutic target in stroke.^19^ Our previous studies showed that SDF‐1a acts not only by binding to CXCR4 but also by binding to receptor CXCR7 to exert a biological effect.^20^ In addition, recent evidence suggests that CXCR7 is a key regulator of organ injury after I/R.^21^ In conclusion, the CXCR7 signaling axis may be involved in cerebral I/R repair.

Recently, several studies have shown that microRNAs (miRNAs) play positive roles in regulating cell survival, inflammation, and apoptosis in response to I/R injury.^22^, ^23^ They are also used in therapeutic interventions and are promising biomarkers.^24^ Moreover, evidence suggests that chemokines mediate miRNA expression and that miRNAs target chemokine signal transduction.^25^, ^26^, ^27^, ^28^, ^29^, ^30^ Despite these findings, the effect of CXCR7 on miRNA expression in I/R brain tissue has not been investigated.

We hypothesized that in the context of cerebral I/R injury, CXCR7 might disrupt and regulate target genes by affecting the expression of miRNAs in ischemic penumbral brain tissue, thus promoting the I/R injury response and playing a protective role in the brain. In this study, we knocked down CXCR7 expression in the brain tissue of MCAO rats using short hairpin RNA against CXCR7. Differentially expressed miRNAs were identified using miRNA microarray to identify direct target genes, and downstream signaling pathways were analyzed. Our results provide new insights into the progression of brain I/R injuries.

## 2 METHODS

### 2.1 Ethics, participants, and animals

The protocol for animal experiments in this study was approved by the Animal Welfare and Ethics Committee of the Wannan Medical College (Approval No. 2021–12). Patients with AIS were recruited from the Department of Neurology, Yijishan Hospital, Wannan Medical College, Anhui Province. Healthy control participants were recruited among individuals undergoing a wellness examination. Additional details are provided in the Appendix S1.

Male Sprague–Dawley (SD) rats (220–240 g) were purchased from Beijing Sibafu Biotechnology Co., Ltd. All rats were fed for 1 week on a normal day and night schedule and were given free access to food and water. The mean body weight of the rats was 240–260 g, the room temperature was 25°C, and the humidity was at 60–70%. The experiments were performed in a blinded manner, and all the rats were subjected to surgery performed by the same surgeon.

### 2.2 Cerebral ischemia/reperfusion model (MCAO)

Cerebral I/R injury was induced by MCAO as previously described.^31^ Briefly, the animals were anesthetized with 2% isoflurane, and analgesia with 4% lidocaine gel was applied to the wound margins. Focal ischemia was induced using thread embolization. First, the right common carotid artery and external carotid artery were exposed and the internal carotid artery was occluded with a microvascular clamp. An embolus coated with silicone rubber (Beijing Sunbio Biotech Co. Ltd., 2634‐20A5, Beijing, China) was inserted through the right external carotid artery, and the middle cerebral artery was bifurcated to induce MCAO. The Zea‐Longa scoring method was used to determine whether occlusion was successfully established (Table S1). Two hours after the occlusion was established, the thread was withdrawn to initiate the reperfusion. Similar operations were performed on sham rats, except for embolus insertion and withdrawal. Body temperature was maintained at 37°C using a servo‐controlled heating blanket throughout surgery. Additional details are provided in the Appendix S1.

### 2.3 Cell sources and culture

HT22 cells were obtained from Millipore (Massachusetts, USA), while C6 cells were obtained from the Chinese Academy of Sciences (Shanghai, China). The cells were cultured in Dulbecco's modified Eagle's medium (DMEM; Hyclone, South Logan, UT, USA) containing 10% fetal bovine serum (FBS; Newseum, Australia), 1% 100 μg/ml streptomycin, and 100 units/ml penicillin (Beyotime, Shanghai, China).^32^, ^33^ Cells were placed in a temperature chamber at 37°C with 5% CO_2_. Normally growing cells were passaged when they reached 80–90% density. Logarithmically grown cells were selected for subsequent experiments.

### 2.4 Oxygen and glucose deprivation (OGD) reperfusion model

As previously described,^34^ C6 cells were placed in a tri‐gas incubator (Thermo Fisher Scientific, Waltham, MA, USA) in which oxygen was replaced with nitrogen. Glucose‐free DMEM was used for 6 h of cell culturing. The original medium was replaced, and the cells were incubated under normal cell culture conditions for 24 h.

### 2.5 Lentivirus microinjection

CXCR7 shRNA lentivirus (5 μl, 10^9^ viral genomes/μl) (Gene Pharma Co. Ltd., Shanghai, China) was microinjected into the right lateral ventricle of SD rats. Microinjection coordinates were as follows: 0.8 mm behind the bregma and 1.8 mm lateral from the sagittal midline at a depth of 4 mm below the skull surface. The needle was then removed slowly. The wound was closed with bone wax and MCAO models were successfully established 14 days later.

### 2.6 Modified Neurological Deficit Score (mNSS)

The mNSS was used to evaluate the neurobehavioral outcome at 6 h, 12 h, 1, 3, and 7 days after MCAO, as described in a previous study.^35^ Each animal was evaluated blindly by two examiners. A more comprehensive description of the mNSS test is provided in Table S2.

### 2.7 2,3,5‐triphenyltetrazolium chloride (TTC) staining

We killed five rats from each group to assess infarct volume at 24 h post‐reperfusion. Then, both sides of 2‐mm thick brain slices were soaked for 15 to 20 min in a 2% 2,3,5‐triphenyltetrazolium chloride solution (T8877, Sigma Aldrich, Germany) at 37°C. Areas without red staining were considered infarcted. Image‐Pro Plus 6.0 was used to capture the infarcted region in each brain slice in its entirety.

### 2.8 Brain water content

The rats were beheaded 24 h after MCAO establishment under deep anesthesia. The brain was divided into the left brain (contralateral), right brain (ipsilateral), and cerebellum. The tissue samples were weighed and then dried at 100°C for 24 h. Brain water content = (wet weight ‐ dry weight)/wet weight × 100%.

### 2.9 Bioinformatics analysis

To observe the effects of CXCR7 expression on miRNA expression in the ischemic penumbra brain tissues of MCAO rats, the brain tissues were divided into two groups: MCAO (*n* = 3) and shRNA‐CXCR7 + MCAO (*n* = 3). Brain tissue (50–100 mg) was removed from ice and washed with PBS. The samples were frozen at −80°C as soon as possible after collection for microfluidic miRNA chip analysis, followed by bioinformatics analysis. The miRNA microarray was performed by a service provider (Aksomics Co. Ltd., Shanghai, China). After quality inspection and quantification, a library was established using a NanoDrop ND‐1000 spectrophotometer (Thermo Fisher Scientific, Waltham, MA, USA), and 50 cycles of sequencing were performed on an Illumina Next Seq 500 sequencer. Cross‐talk quantification of known miRNA and newly discovered miRNA projections based on all trimmed reads was performed using miRDeep2 software. Based on CPM‐standardized miRNAs, the R software, Edge R was used for differential expression calculation, and differential miRNAs were selected. A miRNA cluster map was constructed. The 10 most enriched miRNA target genes were analyzed using miRNA target gene database data, and gene ontology (GO) and pathway analyses of the target genes were performed.

### 2.10 Western blot

Rat brain proteins were isolated by 10% sodium dodecyl sulfate‐polyacrylamide gel electrophoresis and transferred to a BioTrace™ NT nitrocellulose transfer membrane (#66485, Pall, CO, New York, USA). The membrane was blocked at room temperature for 2 h with 5% skim milk or bovine serum albumin and then incubated overnight at 4°C with the following primary antibodies: anti‐CXCR7 (1:100, rabbit, NBP2‐24779, Novus Biologicals, Littleton, CO, USA), anti‐TCF7L2 (1:500, rabbit, A19548, ABclonal, Wuhan, China), anti‐phospho‐TAZ resistant (Ser89) (1:1000, rabbit, 59971 S, Cell Signaling Technology, Danvers, MA, USA), anti‐phospho‐YAP (Ser127) (1:1000, rabbit, 13,008 T, phospho‐Yap (Ser127), Cell Signaling Technology, Danvers, MA, USA), anti‐β‐actin (1:500, rabbit, AF5006, Beyotime, Shanghai, China), and anti‐GAPDH (1:2000, AF1186, Beyotime, Shanghai, China). After washing with TBST three times, the membrane was incubated with secondary antibodies for 12 h and detected with a chemiluminescence HRP substrate solution (#WBKLS0050, Merck‐Millipore, Burlington, MA, USA). Blotting was analyzed using a ChemiDoc XRS system (Bio‐Rad, Hercules, California, USA). The ImageJ software was used to quantify the gray values of the protein fragments. For the loading control, all values were normalized to those of β‐actin or GAPDH, which was used as an internal control.

### 2.11 Quantitative real‐time‐polymerase chain reaction (qRT‐PCR)

Total RNA was extracted from C6 cells using the TRIzol reagent and quantified by spectrophotometry. For miRNA detection, 1 μg of total RNA was reverse‐transcribed in the presence of a stem ring and amplified using a Bulge‐Loop TM miRNA qRT‐PCR Starter Kit (C10211‐2, RIBOBIO Biotechnology, Guangzhou, China). The most stably expressed U6 was used as the endogenous control. Primers for the miRNAs and U6 were purchased from RiboBio Biotechnology. PCR was performed according to the manufacturer's instructions.

For mRNA analysis, 400 ng of total RNA was reverse‐transcribed to cDNA and amplified using a QuantiNova SYBR Green PCR kit (#208054, Qiagen, Hilden, Germany). Primers used for the genes were as follows:

Human CXCR7, forward: 5′‐TCTGCATCTCTTCGACTACTCA‐3′ and reverse: 5′‐GTAGAGCAGGACGCTTTTGTT‐3′;
Human GAPDH, forward: 5′‐GAGAAGTATGACAACAGCCTCAA‐3′ and reverse: 5′‐ GCCATCACGCCACAGTTT‐3′.

### 2.12 Immunofluorescence

The staining of tissue sections and quantification of the staining were performed as we had previously described.^36^ Cultured cells and tissue sections were fixed in paraformaldehyde (4%), followed by permeabilization with Triton X‐100 (0.1%) in PBS for 30 min, and blocked with goat serum (3%) for 2 h. Brain slices were incubated at 4°C overnight with the following primary antibodies: anti‐NeuN (1:200, mouse, 94403 S, Cell Signaling Technology, Danvers, MA, USA) and anti‐CXCR7 (1:200, rabbit, NBp2‐24,779, Novus Biologicals, Littleton, CO, USA). The slides were washed with PBS and incubated with DyLight 594‐conjugated goat anti‐rabbit IgG (1:500, E032420‐01, EarthOx, San Francisco, CA, USA) or DyLight 488‐conjugated goat anti‐mouse IgG (1:500, E032210‐01, EarthOx, San Francisco, CA, USA) in the dark for 2 h at room temperature. After staining with DAPI (P0131‐25 ml, Beyotime, Shanghai, China), all slides were read and imaged using a confocal microscope (LSM800, ZEISS, Germany).

### 2.13 Dual luciferase activity assays

C6 cells were transfected with the pMIR REPORT luciferase vector with the wild‐type or mutated TCF7L2‐untranslated region (UTR; Hanbio, Guangzhou, China) for 24 h. Dual luciferase reporter assays were performed following the manufacturer's protocol (E2920, Promega, Madison, WI). To examine whether miR‐182 directly regulated TCF7L2 expression, a predicted target of miR‐182, pSI‐Check2 dual‐luciferase miRNA target expression vector (Hanbio Biotechnology, Wuhan, China), was used. The partial 3′‐UTR of TCF7L2 from rat genomic DNA was amplified by PCR and cloned into the pSI‐Check2 vector to produce a wild‐type (Wt) reporter. One mutant (Mut) reporter was generated by site‐directed mutagenesis within the miR‐182 seed‐match region. Plasmid construction and dual‐luciferase kits were purchased from Hanbio Biotechnology. The pSI‐Check2‐TCF7L2‐3′UTR (Wt or Mut) was cotransfected into C6 cells either with vehicle (Control), miRNA negative control oligonucleotide (miRNA NC), or miR‐182 mimic using Lipofectamine™ 3000 (L3000001, Thermo Fisher Scientific, Waltham, MA, USA), according to the transfection procedures. Cell lysates were harvested 24 h after transfection, and dual‐luciferase reporter activity was measured using a kit (Hanbio Biotechnology, Wuhan, China) and multimode microplate reader (Promega GloMax‐Muti®, Promega, Madison, WI, USA).

### 2.14 Statistical analysis

All data are presented as mean ± SEM. Statistical analyses were performed using the GraphPad Prism version 7. Data were tested for Gaussian distribution using the ShapiroWilk test and then analyzed by *t*‐test, one‐way ANOVA, or two‐way ANOVA according to the situation as appropriate. Data that did not exhibit a normal/Gaussian distribution were analyzed via a non‐parametric equivalent and used by the post‐hoc test for pairwise comparison of the data. Results were considered significant at *p* < 0.05.

## 3 RESULTS

### 3.1 CXCR7 was upregulated in the MCAO rat ischemic penumbra brain tissue, OGD‐induced C6 cells, and patients with acute ischemic stroke undergoing mechanical thrombectomy

**FIGURE 1:**
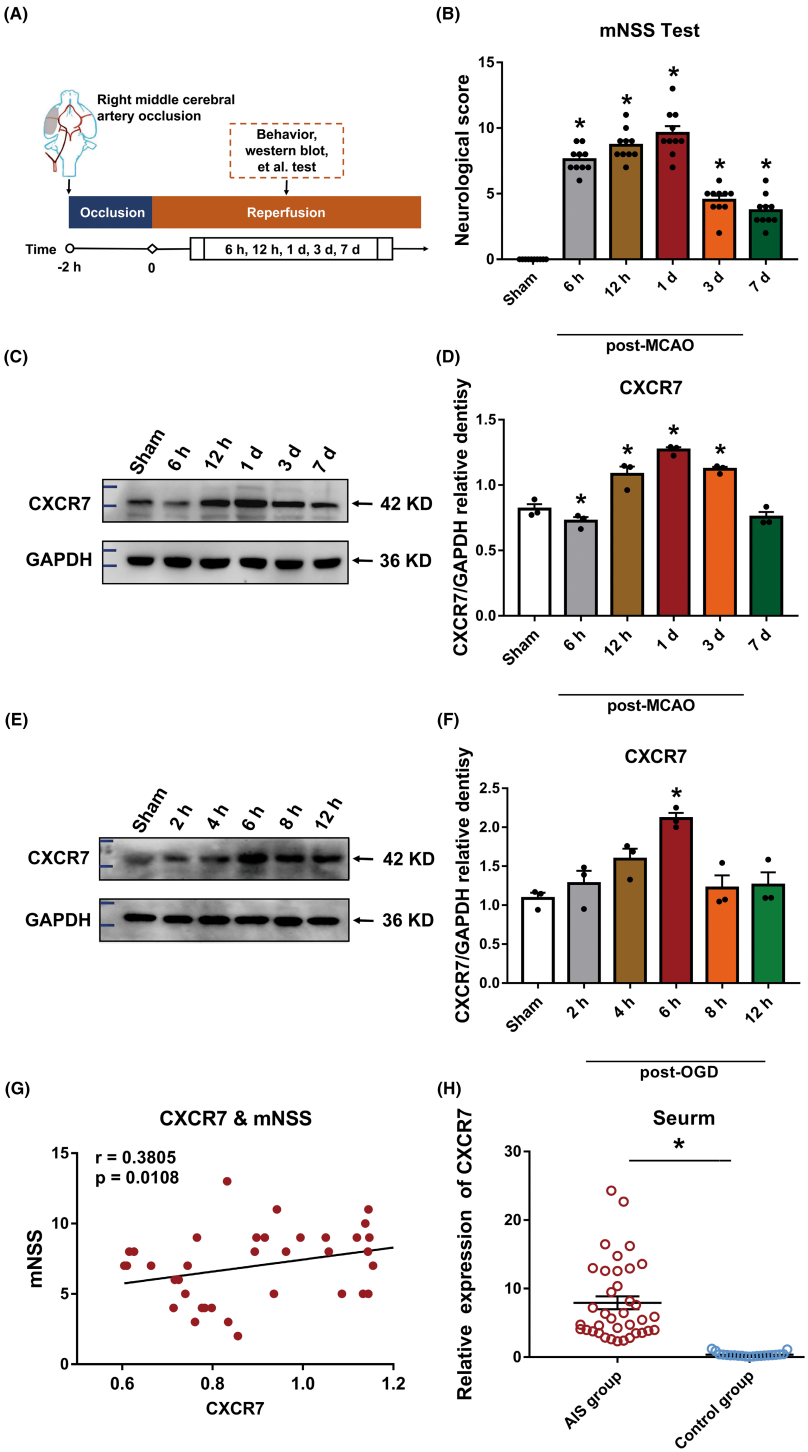
CXCR7 expression is upregulated in middle cerebral artery occlusion (MCAO) rat ischemic penumbra brain tissue, in C6 cells under oxygen and glucose deprivation (OGD), and in the peripheral blood of a patient with acute ischemic stroke with thrombectomy. (A) Experimental procedure and timeline. (B) The modified neurological severity score (mNSS) following MCAO establishment. (C) Relative gene expression of CXCR7 in the ischemic penumbra brain tissues of the rats at 6 h, 12 h, 1, 3, and 7 days after MCAO establishment and in the sham group (*n* = 3). (D) Densitometric quantification of CXCR7/GAPDH. (E) Relative gene expression of CXCR7 in the C6 cells at 0, 2, 4, 6, and 8 h under hypoxia condition and in the sham group (*n* = 3). (F) Densitometric quantification of CXCR7/GAPDH. (G) Correlation between CXCR7 expression and mNSS scores using Pearson's correlation coefficient. (H) Levels of CXCR7 are increased in the serum of patients with acute ischemic stroke undergoing mechanical thrombectomy (*n* = 17) compared with that in healthy control participants (*n* = 10). *p < 0.05 versus the sham group

Western blotting was performed to detect CXCR7 protein expression in the ischemic penumbra at different time points after I/R (Figure 1A). The results showed that CXCR7 protein expression began to decrease at 6 h, then gradually increased, peaking on day 1, and then gradually decreased and returned to normal levels in the ischemic penumbra after I/R injury. Compared with the normal group, CXCR7 protein expression was significantly decreased within 6 h and increased in the 12‐h, 1‐, 3‐, and 7‐day groups (all, *p* < 0.05) (Figure 1C,D, Figure S13). Neurological deficit scores indicated that neurological function had deteriorated noticeably after I/R. The mNSS test score increased to a maximum at 24 h post‐MCAO establishment (*p* < 0.05) (Figure 1B). Thus, we selected 24 h as the time point to elucidate the mechanism of CXCR7‐mediated miRNA expression after I/R injury. A linear regression analysis revealed that 24 h after surgery, patients who had higher CXCR7 expression and higher NIHSS scores showed more severe symptoms of neurological deficit (Figure 1G). To determine whether CXCR7 was involved in I/R, we examined CXCR7 levels in the peripheral blood of healthy control participants and patients with AIS undergoing mechanical thrombectomy. Infarct volume was measured using 3.0 T magnetic resonance imaging diffusion‐weighted imaging sequences within 24 h after admission (Figure S1). The demographic and clinical characteristics of the participants are listed in Table S3. Further analysis of the clinical samples showed that the CXCR7 protein level was significantly increased in the serum of patients with AIS 24 h after surgery compared to that in the sex‐ and age‐matched normal control participant serum by qRT‐PCR (Figure 1H). This was also found in the OGD‐induced C6 cells upregulation of CXCR7 protein levels. Specifically, CXCR7 was markedly induced at early 2 h in response to OGD, peaked at 6 h, and was maintained with a slight decrease until 12 h (Figure 1E,F, Figure S13).

### 3.2 Colocalization of CXCR7 and NeuN, GFAP, and Iba1 after cerebral I/R injury

**FIGURE 2:**
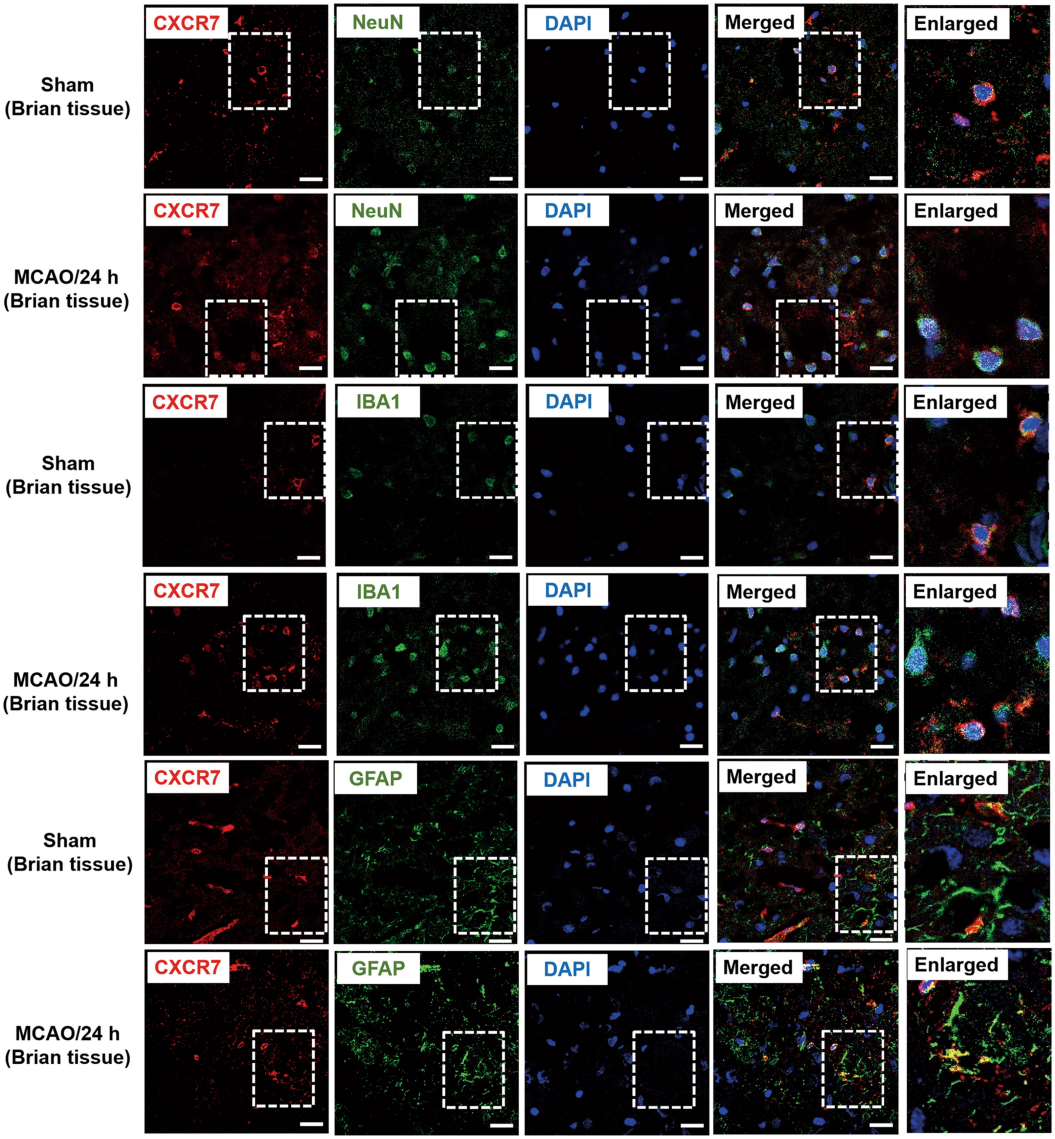
CXCR7 is expressed in neurons, microglia, and astrocytes. Co‐localization of CXCR7 with NeuN (A), Iba1 (B), and GFAP (C) after sham, MCAO, or OGD. Representative images of immunofluorescence staining showing the expression of CXCR7 (red), NeuN (green), Iba1 (green), and GFAP (green), including areas in which they overlap, shown in rectangles. Scale bar: 20 μm. Brain tissue samples were obtained from the ischemic penumbra area 24 h after MCAO (*n* = 3)

Double‐label fluorescence immunohistochemistry was performed to examine the phenotypes of CXCR7‐positive cells in the brains of MCAO rats. We found that CXCR7 was localized to neurons, microglia, and astrocytes in the brains of rats with MCAO and normal brain tissues. CXCR7 with NeuN, GFAP, and Iba1 in the MCAO group showed enhanced fluorescence intensity on the cell membrane compared to that in the sham group. In the sham group, the fluorescence signals of CXCR7 with NeuN, GFAP, and Iba1 were primarily located on the cell membrane. Notably, this colocalization was detected in the cytosol around the nucleus post‐MCAO (Figure 2, Figure S2). Meanwhile, we also made localization in C6 cells and obtained similar results (Figures S3 and S4).

### 3.3 Knockdown of CXCR7 expression aggravated ischemia/reperfusion injury in MCAO rats

**FIGURE 3:**
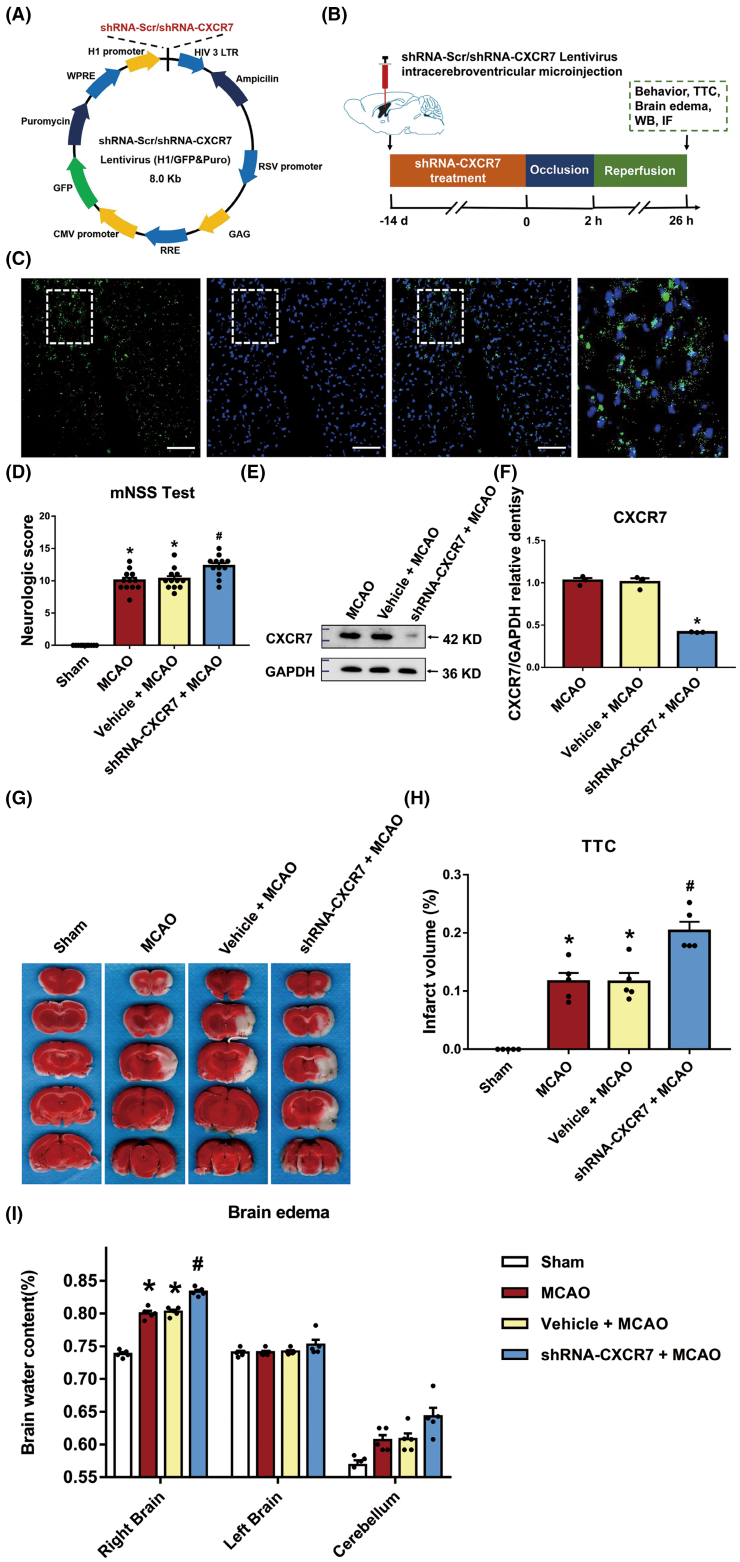
Knocking down CXCR7 expression aggravates ischemia/reperfusion (I/R) injury in MCAO rats. (A) Schematic showing the lentivirus vector encoding CXCR7. (B) Experimental procedure and timeline. (C) Representative images of the rat lateral ventricle microinjected with GFP lentiviruses. Rats were killed 2 weeks after microinjection, and GFP expression was measured. Scale bar = 100 μm. (D) Effects of CXCR7 lentivirus microinjection on neurological behaviors of the MCAO rats (*n* = 12). (E) Representative western blot bands showing CXCR7 expression in the MCAO, vehicle + MCAO, and shRNA‐CXCR7 + MCAO groups. (F) Densitometric quantification of CXCR7/β‐actin. (G) TTC‐stained brain sections and (H) quantitative data illustrating the increased infarct volume after CXCR7 knockdown (*n* = 5). (I) Brain edema 24 h after surgery in the sham, MCAO, vehicle + MCAO, and shRNA‐CXCR7 + MCAO groups (*n* = 5). The brain was divided into three parts: The right brain, left brain, and cerebellum. *p < 0.05 versus the sham group, #*p* < 0.05 versus the MCAO group

Next, we investigated the effect of CXCR7 knockdown on I/R rats by microinjecting either shRNA‐Scr‐GFP or shRNA‐CXCR7‐GFP lentivirus into the rat lateral ventricle, as illustrated in Figure 3A,B. First, we selected and verified the lentivirus (Figure S5). Two weeks after the injection, the expression of green fluorescent protein (GFP) lentivirus was largely restricted to the whole brain (Figure 3C), and CXCR7 expression was significantly downregulated in the brain tissues (Figure 3E,F, Figure S13). shRNA‐CXCR7 treatment considerably worsened neurological function compared to that in the untreated MCAO group, as evidenced by the results of the mNSS test (*p* < 0.05) (Figure 3D). After TTC staining of four corresponding coronal brain sections from sham, MCAO, vehicle + MCAO, and shRNA‐CXCR7 + MCAO rats killed 24 h after MCAO establishment, the infarcted areas (white) appeared larger in the shRNA‐CXCR7‐treated rats, which was confirmed by the infarct volumetry results (*n* = 5) (*p* < 0.05) (Figure 3G,H). In addition, brain water content in the ipsilateral hemisphere was significantly elevated following MCAO establishment (*p* < 0.05) (Figure 3I). The shRNA‐CXCR7 + MCAO group displayed higher brain water content in the ipsilateral hemisphere than the MCAO group (*p* < 0.05) (Figure 3I). There were no significant differences in neurobehavioral function or brain water content between the MCAO and vehicle + MCAO groups (*p* > 0.05). shRNA‐CXCR7 treatment further increased cerebral edema and infarct volume in the experimental groups compared with that in the sham group; the neurological deficits were significantly exacerbated by knockdown of CXCR7 expression. However, there were no significant differences in these functional experiments between the shRNA‐CXCR7 + MCAO and shRNA‐CXCR7 + AMD3100 + MCAO groups (*p* > 0.05) (Figures S6–S9 and S13).

### 3.4 Knockdown of CXCR7 expression induced differential expression of miRNAs in the ischemic penumbra of MCAO rats

**FIGURE 4:**
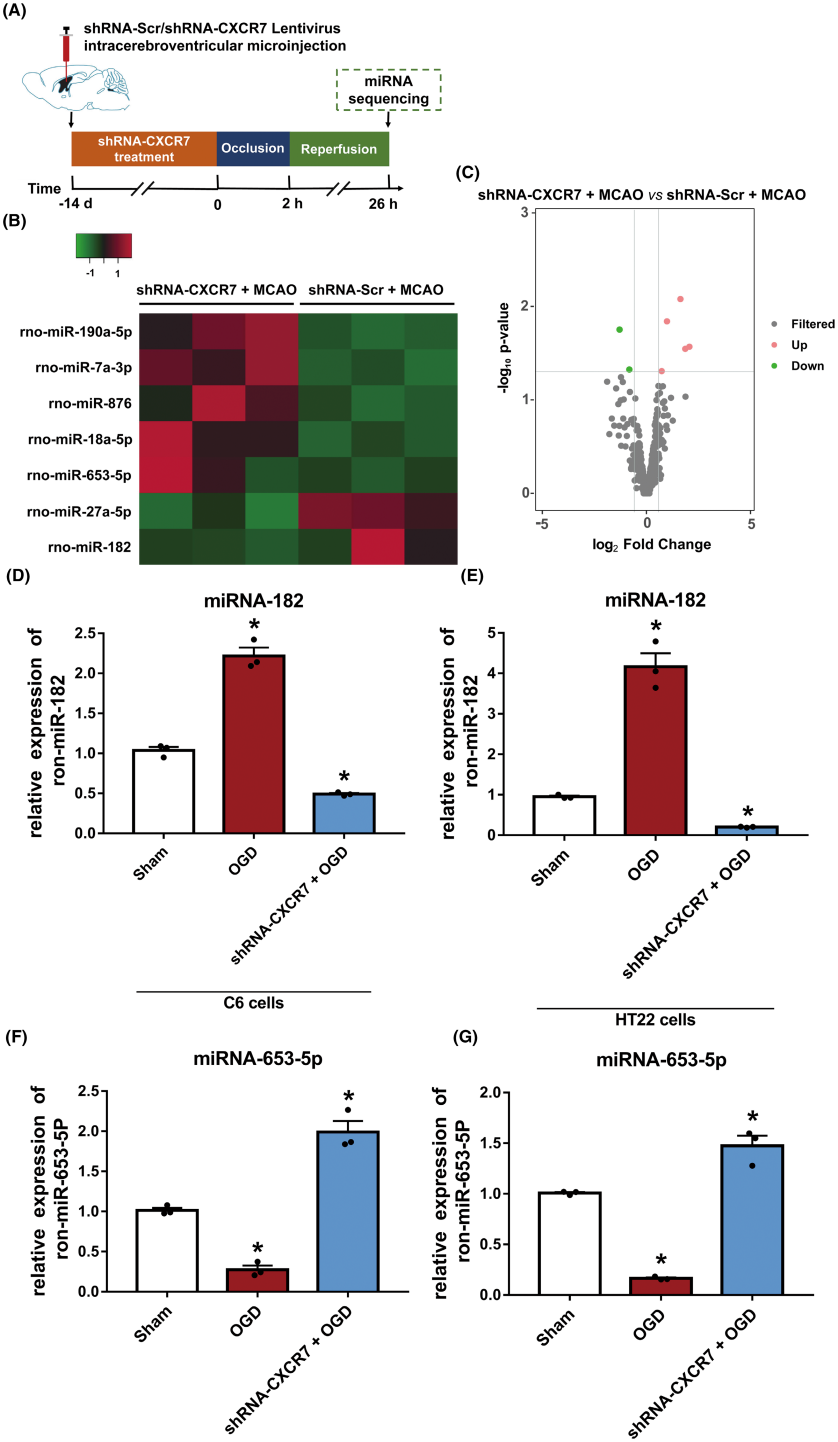
Knocking down CXCR7 expression induces differential expression of miRNAs in the ischemia penumbra of MCAO rats. (A) Experimental procedure and timeline. (B) Distinct miRNA expression in the cerebral tissue of ischemic penumbra in the MCAO rats in which CXCR7 expression was knocked down compared with that in control tissues (fold change ≥1.5, *p* < 0.05). Red represents increased gene expression; green represents decreased expression. (C) Significant up‐ and downregulation of miRNA expression (fold change ≥1.5). Total RNA was extracted from three control and three MCAO rat ischemic penumbra brain tissues, and miRNA sequence analysis was performed after purification. (D) Levels of miR‐182 decreased by qRT‐PCR in C6 cells and HT22 cells (E) after OGD with shRNA‐CXCR7 treatment (*n* = 3) compared with those in the sham group (*n* = 3). (F) The level of miR‐653‐5P detected by qRT‐PCR in C6 cells and HT22 cells (G) after OGD with shRNA‐CXCR7 treatment (*n* = 3) compared with those in the sham group (*n* = 3). *p < 0.05 versus the sham group

Using an miRNA microarray assay, we compared the miRNA expression profile of ischemic penumbra brain tissues after CXCR7 knockdown with that in control ischemic penumbra brain tissues without CXCR7 treatment (Figure 4A). A total of seven miRNAs were found to be significantly differentially expressed between the shRNA‐NC + MCAO and shRNA‐CXCR7 + MCAO groups, and five miRNAs were upregulated and two miRNAs were downregulated after shRNA‐CXCR7 treatment (threshold difference >1.5‐fold, *p* ≤ 0.05, and mean counts per million reads value ≥1) (Figure 4B,C and Table S4). miR‐653‐5p was the most upregulated miRNA, with a fold change of 4.19, and miR‐182 was the most downregulated miRNA, with a fold change of 0.41 (Figure S11). Next, we used an OGD in vitro model to simulate an isolated cerebral ischemia model for further verification. The expression of miR‐653‐5p and miR‐182 was also measured by qRT‐PCR in C6 and HT22 cell lines, and the results showed patterns of down‐ and upregulation similar to those shown in the results of the miRNA microarray approach (Figure 4D–G). Furthermore, there were no significant differences in miR‐182 and miR‐653‐5p expression between the shRNA‐CXCR7 + OGD and shRNA‐CXCR7 + AMD3100 + OGD groups (*p* > 0.05) (Figure S10).

### 3.5 miR‐182 directly targets the TCF7L2 gene by interacting with the 3′ UTR

**FIGURE 5:**
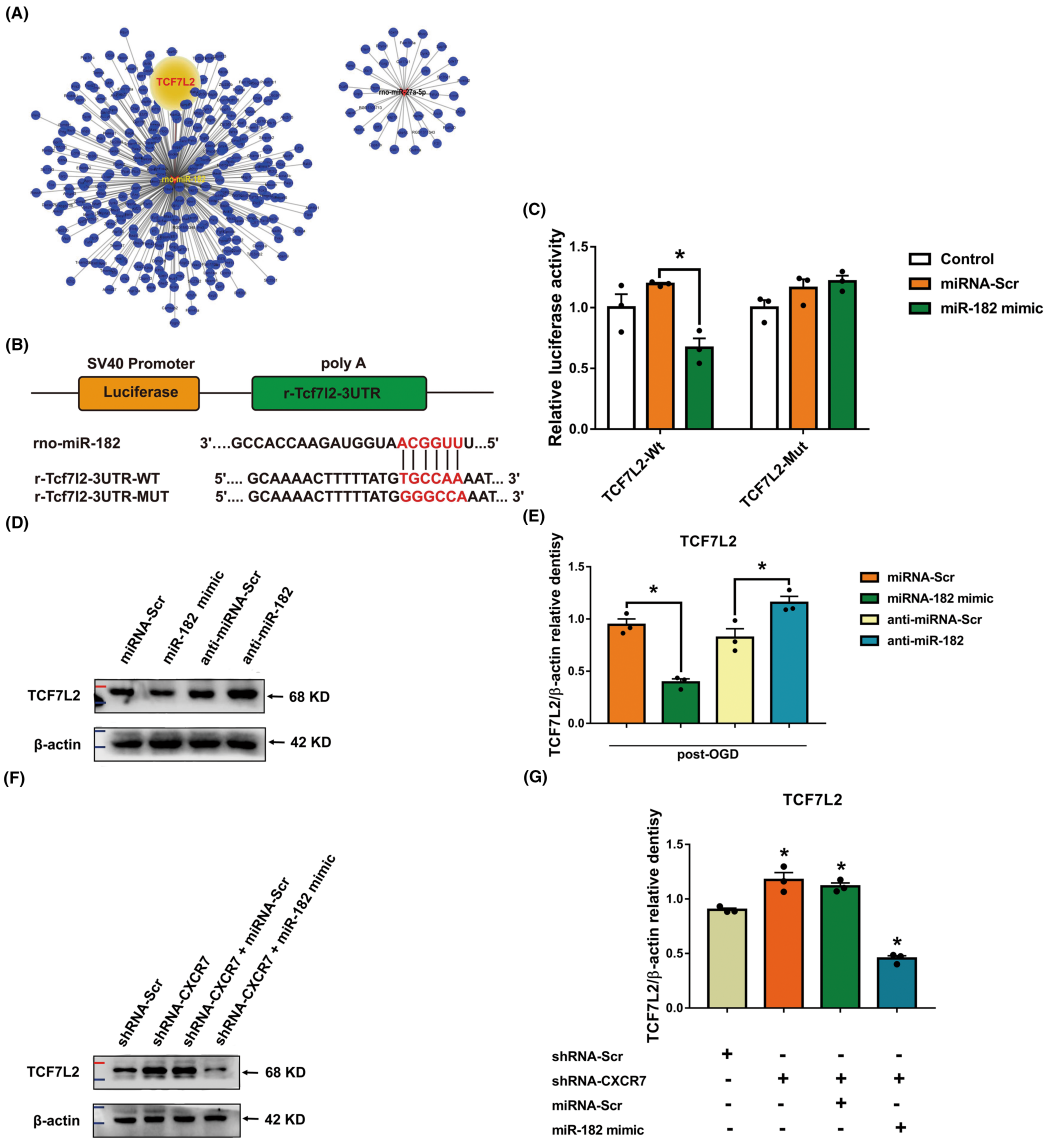
The CXCR7/miR‐182 axis affects ischemic/reperfusion (I/R) progression mediated through TCF7L2. (A) All downregulated miRNA gene regulatory networks. (B) Putative miR‐182 binding sequence in the 3′ untranslated region (UTR) of TCF7L2 mRNA. A mutation was generated in the TCF7L2 3′UTR sequence at the complementary site of the seed region in miR‐182. A TCF7L2 3′UTR fragment with a wild‐type (TCF7L2‐Wt) or a mutant (TCF7L2‐Mut) miR‐182‐binding sequence was cloned downstream of the luciferase reporter gene. (C) The TCF7L2‐Wt or TCF7L2‐Mut reporter vector cotransfected into C6 cells with vehicle (control), miR‐182 mimic, or negative control (miRNA‐Scr). The normalized luciferase activity in the control group was set as the relative luciferase activity. The data are presented as the mean ± standard error of the mean (*n* = 3). (D) Representative western blot bands showing TCF7L2 expression in the miRNA‐Scr, miR‐182 mimic, anti‐miRNA‐Scr, and anti‐miR‐182 groups of C6 cells (*n* = 3). (E) Densitometric quantification of TCF7L2/β‐actin. (F) Representative western blot bands showing TCF7L2 expression in the shRNA‐Scr, shRNA‐CXCR7, shRNA‐CXCR7 + miRNA‐Scr, and shRNA‐CXCR7 + miR‐182 mimic groups of C6 cells. (G) Densitometric quantification of TCF7L2/β‐actin. *p < 0.05 versus the miRNA‐Scr group

Based on the microarray screening and bioinformatics analysis of potential target genes (Figure 5A and Figure S12), miR‐182, a miRNA significantly downregulated by shRNA‐CXCR7, was selected for further study. We sought to identify the direct target of miR‐182 that mediated I/R injury. We used the TargetScan7.2 and miRDB databases to predict the potential targets of miR‐182 (Figure S12) and selected candidates with a percentage higher than 0.6 or miRDB scores higher than 70. Using the miRDB and TargetScan databases of predicted miRNA targets, we found that TCF7L2 was a potential target gene of miR‐182. Thus, we used a dual‐luciferase reporter to verify the predictions in C6 cells. The miR‐182‐binding sequences in the 3′ UTR of WT TCF7L2 mRNA (TCF7L2‐Wt) or its mutant (TCF7L2‐Mut) were subcloned downstream of the firefly luciferase reporter gene in a pSI‐Check2 vector (Figure 5B). We found that the miR‐182 mimic significantly inhibited the luciferase activity of the TCF7L2 3‐′UTR reporter but did not affect the luciferase activity of the TCF7L2 3‐UTR reporter with mutated miR‐182‐binding sites. Moreover, there was no change in luciferase activity when an miRNA‐negative control was cotransfected with either the WT or the reporter construct (Figure 5C). These results suggest that TCF7L2 is a target of miR‐182. To confirm that TCF7L2 was a downstream target of CXCR7‐mediated I/R progression, we performed subsequent functional rescue assays. We first transfected C6 cells with a miR‐182 mimic or an miR‐182 inhibitor and their respective controls and verified the construct expression efficiency by qRT‐PCR and western blotting (Figure 5D,E, Figure S13). Moreover, by separately transfecting shRNA‐CXCR7 and shRNA‐CXCR7 + the miR‐182 mimic into C6 cells, we found that the protein levels of TCF7L2 increased and decreased, respectively (Figure 5F,G, Figure S13). These data suggest that direct posttranscriptional regulation of TCF7L2 is mediated through the CXCR7/miR‐182 axis.

### 3.6 CXCR7 expression activated the Hippo pathway

**FIGURE 6:**
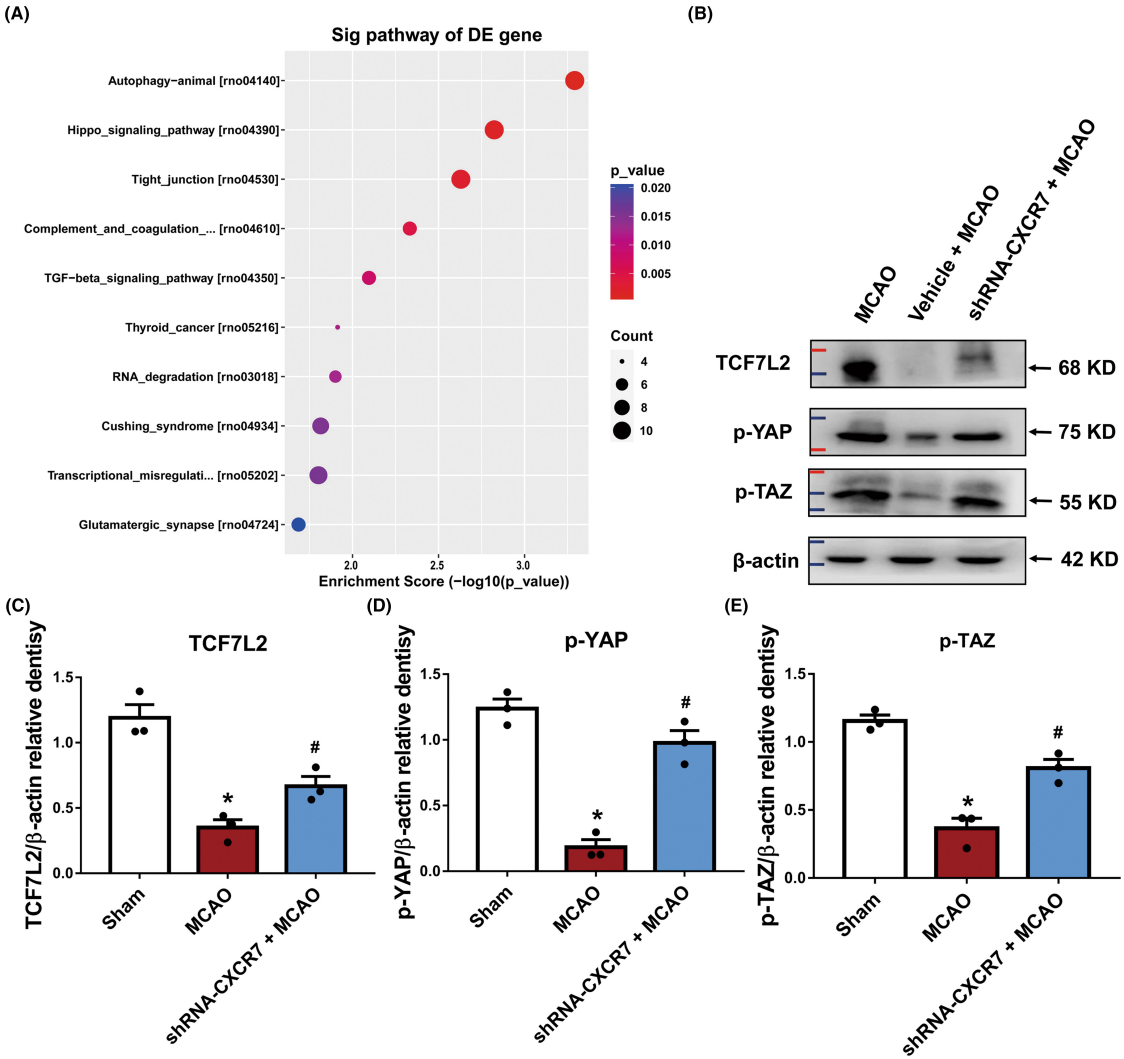
CXCR7 plays a protective role in the brain‐mediated Hippo signaling pathway. (A) The 10 most significantly enriched ischemia/reperfusion (I/R)‐associated pathways with downregulated CXCR7 expression. (B) Representative protein expression levels of TCF7L2, p‐YAP, and p‐TAZ detected at 24 h following surgery in the sham, MCAO, and shRNA‐CXCR7 + MCAO groups (*n* = 3). (C) Densitometric quantification of TCF7L2/β‐actin. (D) Densitometric quantification of p‐YAP/β‐actin. (E) Densitometric quantification of p‐TAZ/β‐actin. *p < 0.05 versus the sham group, ^#^ *p* < 0.05 versus the MCAO group

The Kyoto Encyclopedia of Genes and Genomes (KEGG) pathway analysis of the predicted target genes revealed that the two most enriched KEGG pathways in shRNA‐CXCR7 were the autophagy and Hippo pathways (Figure 6A). After referring to a previous report,^37^ we performed bioinformatics analysis to identify the potential substrates of TCF7L2 and found YAP and TAZ proteins to be of potential interests. Therefore, we selected YAP and TAZ for further experiments. Next, we explored potential downstream effectors of TCF7L2 activation in CXCR7‐mediated I/R injury. We detected the expression of YAP and TAZ, the main effector molecules in the Hippo pathway, and found that in ischemic penumbra brain tissue treated with shRNA‐CXCR7, phosphorylation of YAP at Ser127 and TAZ at Ser89 was dramatically increased (Figure 6B,D,E, Figure S13), whereas its expression was decreased in the MCAO group. Furthermore, in the ischemic penumbra brain tissue of the sham and shRNA‐CXCR7 + MCAO groups, the levels of phosphorylated Ser127‐YAP and Ser89‐TAZ were increased, and this increase was accompanied by an increase in TCF7L2 expression (Figure 6B,C). Importantly, we did not find a relatively high positive correlation between CXCR7 and miR‐182; in contrast, we found a negative correlation between CXCR7 and TCF7L2, phospho‐Ser127‐YAP, and phospho‐Ser89‐TAZ (Figure 6B–E). Hence, the loss of CXCR7 expression resulted in the abnormal activation of miR‐182, and low expression of miR‐182 led to the disruption of the expression of the downstream target gene TCF7L2 and subsequent aggravation of I/R injury via the induced phosphorylation of YAP at Ser127 and TAZ at Ser89.

## 4 DISCUSSION

**FIGURE 7:**
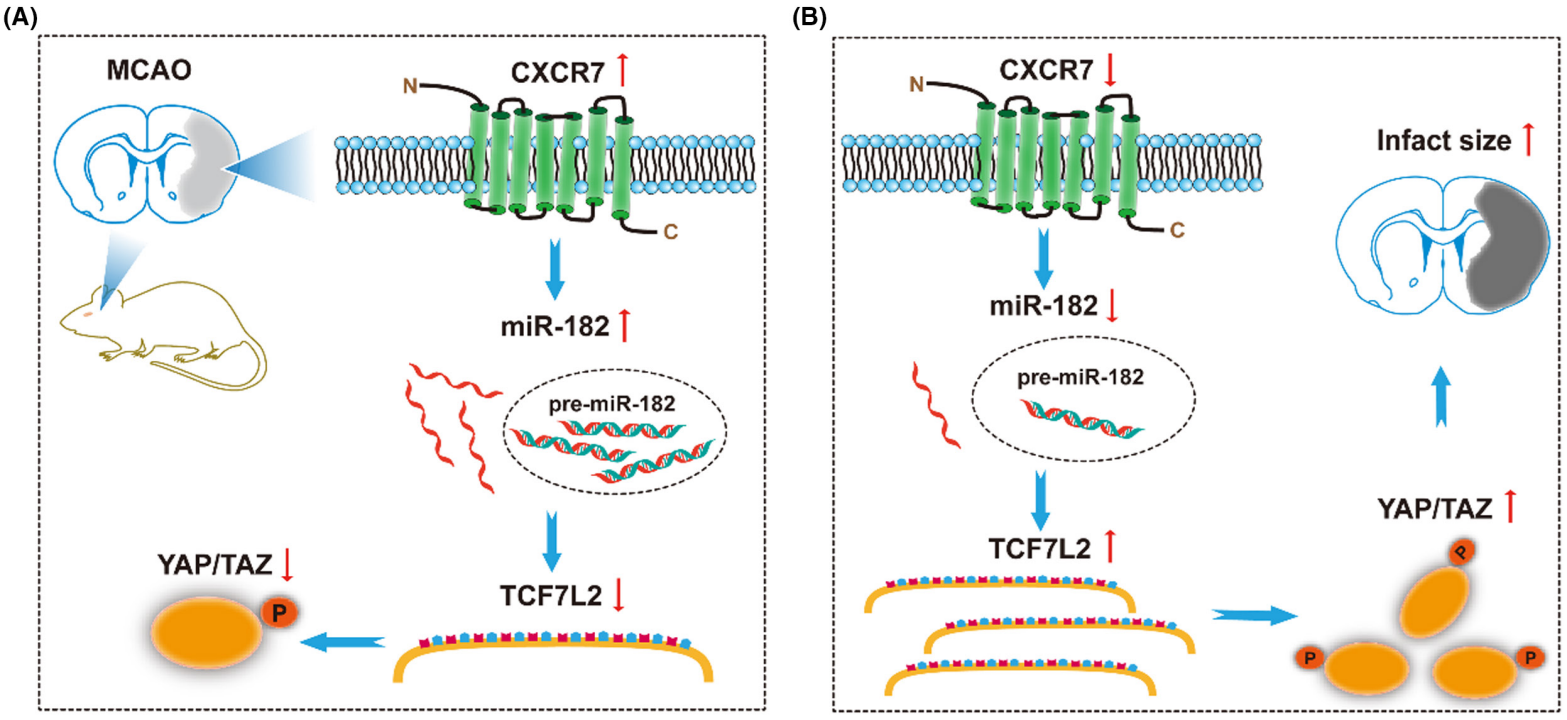
Schematic illustration of CXCR7 ameliorating ischemia/reperfusion (I/R) injury in acute ischemic stroke. (A) CXCR7 is upregulated in MCAO rats, and miR‐182 promotes pathological process and plays a protective role in the brain by forming an endogenous competitive RNA network with TCF7L2/Hippo. (B) Decreased expression of CXCR7 disrupts the miR‐182 expression, reducing the inhibitory effect of downstream target genes, thereby affecting the activation of Hippo pathway and subsequently aggravating cerebral ischemia reperfusion injury

In this study, in agreement with previously published data, we have demonstrated that patients with stroke express high CXCR7 levels (Figure 1). Due to the clinical relevance of CXCR7 expression in patient prognosis, we extended our understanding of its role in cerebral I/R injury development. CXCR7 is considered a critical regulator of platelet activation, thrombus formation, and organ injury following I/R.^21^ Low CXCR7 expression has been demonstrated to increase infarct volume and deteriorate function in I/R injury models.^15^ In line with our findings, we have seen that low expression of CXCR7 causes more infarct volumes and worse nerve defects (Figure 3). Based on previous research,^29^, ^38^, ^39^ we further established that chemokines play a role in mediating the expression of miRNAs and their downstream pathways (Figures 4, 5, 6). This effect was dependent on CXCR7 cell membrane levels, which showed that CXCR7 was the most responsive at high levels on the cell membrane (Figure 2). Our results support that blocking the CXCR7/miR‐182/TCF7L2/Hippo axis profoundly attenuates I/R injury in the MCAO rat model (Figure 7).

In recent years, CXCR7 has been considered a target for cerebral I/R injury therapy, and therapy involving this pathway is currently being developed.^40^ Despite progress in CXCR7 theories, research on the role of CXCR7 in cerebral I/R injury in epigenetics remains unknown. CXCR7 is expressed on many cell types in the nervous system, including microglia, astrocytes, and neurons,^17^, ^41^, ^42^ and we once again proved (Figure 2) that the changes in CXCR7 are the effect of mixed cell types in brain tissue. Experimental models by our group have established that aggravation of cerebral I/R injury can be achieved in MCAO rats by the knockdown of CXCR7 (Figure 3C,E,F). Silencing CXCR7 increased neurological deficits, infarct volume, and brain edema in MCAO rats (Figure 3D,G–I). Notably, loss of CXCR7 has also been shown to worsen cardiac function, increase infarct size, and increase mortality after myocardial infarction.^15^ These results indicate that CXCR7 can prevent the development of cerebral I/R injury. Although CXCR7 was not successful in completely preventing the occurrence and development of cerebral I/R injury, it is possible that administration of CXCR7 as a preventive treatment could prevent the aggravation of brain injury. In summary, our data indicate that CXCR7 is a crucial factor in the progression of cerebral I/R injury.

In this study, we attempted to screen for differentially expressed miRNAs following CXCR7 knockdown using short hairpin RNA in cerebral I/R models. We found that the miRNA expression profiles of rats subjected to CXCR7 deficiency were significantly differentially expressed compared with those of control rats, and we further predicted the corresponding downstream target genes and pathways (Figures 4A–C, 5A and 6A). Theoretically, the regulation of miR‐182 by CXCR7 would lead us to assume that upregulation of CXCR7 would reduce the levels of the miRNAs. Surprisingly, this was not the case, and CXCR7 from the ischemic penumbra dramatically increased the miR‐182 levels. Furthermore, CXCR7 from different cell lines with OGD, relative to the control cell lines, also increased miR‐182 expression (Figure 4D–G). The target genes TCF7L2 of miR‐182 was selectively reduced in the CXCR7 upregulated cells (Figure 5D,E). Controversially, it was also reported that CXCR4 might be involved. To test the potential involvement of CXCR4 in CXCR7 knockdown, we performed functional experiments with AMD3100^43^, ^44^ directed against CXCR4. CXCR7 expression was not affected by the inhibitor (Figures S6–S9). Furthermore, qRT‐PCR analysis showed that shRNA‐CXCR7‐induced miR‐182 expression was significantly reduced irrespective of the presence of anti‐CXCR4 or AMD3100, whereas shRNA‐CXCR7‐induced miR‐653‐5p expression was significantly increased (Figure S10). Thus, CXCR4 is not required for CXCR7‐dependent miRNAs.

This study identified, for the first time, the regulation of miRNA expression profiles by CXCR7 in cerebral I/R injury. Here, we demonstrated that MCAO rats treated with shRNA‐CXCR7 not only downregulated miR‐182 but also that the latter inhibited their target genes, TCF7L2 (Figure 5D,E). Considering the important function of the Hippo pathway in cerebral I/R injury,^45^ we propose that reducing CXCR7 levels will cause activation of the Hippo pathway cascade to exert brain protection (Figure 7). Furthermore, supporting this opinion, transfection of shRNA‐CXCR7 into MCAO rats increased the expression of TCF7L2, p‐YAP, and p‐TAZ, while MCAO rats abrogated this effect, indicating that the Hippo pathway was mediated by CXCR7 (Figure 6B–E). Interestingly, we not only found that CXCR7 regulated the Hippo pathway, but also revealed the posttranscriptional regulation of this pathway by CXCR7.

These findings emphasize that CXCR7 is a crucial protector in cerebral I/R injury. Our results demonstrated that activation of the miR‐182 pathway by CXCR7 upregulation decreased TCF7L2 expression. Concomitantly and independently of the elevation of miR‐182, Hippo signaling was activated, increasing the phosphorylation of key effector molecules in the Hippo pathway (Figure 6). This was observed in our animal model as well as in other I/R models. Hippo signaling has been shown to be involved in I/R injury, crucial for protection against blood–brain barrier disruption.^45^ In fact, when cerebral I/R injury is induced by MCAO, although miR‐182 levels are elevated, p‐YAP and p‐TAZ levels decrease, emphasizing that these two pathways are regulated by different mechanisms (Figures 5 and 6). These results suggest that miR‐182 pathway activation and Hippo inhibition both have to occur to regulate signaling pathways controlled by CXCR7. In support of this, we found that MCAO rats with CXCR7 expression became desensitized to inhibition by p‐YAP and p‐TAZ, whereas low expression of CXCR7 promoted its phosphorylation (Figure 6). This means that the cells that were previously dependent on the Hippo pathway for their survival were now dependent on CXCR7, thus establishing a brain‐protective dependency on this gene. These results provide a rational basis for CXCR7‐targeted therapy for cerebral I/R injury. Moreover, these results suggest that CXCR7 levels should be considered when treating cerebral I/R injury with miR‐182 target gene inhibitors.

## 5 CONCLUSION

Our results indicate that CXCR7 is a protective factor and acts against cerebral I/R injury by regulating the activation of the downstream target gene TCF7L2, and against the resulting Hippo pathway activation by disrupting the expression profile of miR‐182. Targeting the CXCR7/miR‐182/TCF7L2/Hippo regulatory axis is a novel strategy for interfering with cerebral I/R injury and improving the prognosis of interventional surgery.

## AUTHOR CONTRIBUTIONS

Zhiming Zhou conceived the concept of the project; Yang Xu, Qi Wang, and Zhiming Zhou designed the studies; Qi Wang, Sifan Xu, Bin Wang, Yachen Ji, and Yu Qin performed the experiments. Qi Wang, Sifan Xu, Bin Wang, and Yachen Ji performed initial data analysis. Bin Wang and Qian Yang participated in drawing and typesetting. Qi Wang drafted the manuscript. Zhiming Zhou and Yang Xu performed the final analysis of the data and wrote the manuscript. All authors approved the final manuscript.

## FUNDING INFORMATION

This study was supported by grants from the National Natural Science Foundation of China (Grant No(s). 82170368, 81701161); the Open Project of Key Laboratory of Non‐coding RNA Transformation Research of Anhui Higher Education Institution (Wannan Medical College) (RNA202201); Natural Science Research Project of Anhui Educational Committee (2022AH040180, 2022AH051219); Science and Technology Project of Wuhu City (2022jc63); Wannan Medical College, Anhui, China (YR201802, KGF2019G02).

## CONFLICT OF INTEREST

The authors declare no conflict of interest.

## Supporting information


AppendixS1

AppendixS2

## Data Availability

The data supporting the findings of this study are available from the corresponding authors upon reasonable request.

## References

[1] Kernan WN , Ovbiagele B , Black HR , et al. Guidelines for the prevention of stroke in patients with stroke and transient ischemic attack: a guideline for healthcare professionals from the American Heart Association/American Stroke Association. Stroke. 2014;45(7):2160‐2236. 24788967 10.1161/STR.0000000000000024

[2] Kalkonde YV , Deshmukh MD , Sahane V , et al. Stroke is the leading cause of death in rural Gadchiroli, India: a prospective community‐based study. Stroke. 2015;46(7):1764‐1768. 25999388 10.1161/STROKEAHA.115.008918

[3] Fisher M , Saver JL . Future directions of acute ischaemic stroke therapy. Lancet Neurol. 2015;14(7):758‐767. 26067128 10.1016/S1474-4422(15)00054-X

[4] Nomura E , Kohriyama T , Kozuka K , Kajikawa H , Nakamura S , Matsumoto M . Significance of serum soluble thrombomodulin level in acute cerebral infarction. Eur J Neurol. 2004;11(5):329‐334. 15142226 10.1111/j.1468-1331.2004.00776.x

[5] Allen CL , Bayraktutan U . Oxidative stress and its role in the pathogenesis of ischaemic stroke. Int J Stroke. 2009;4(6):461‐470. 19930058 10.1111/j.1747-4949.2009.00387.x

[6] Salim S . Oxidative stress and the central nervous system. J Pharmacol Exp Ther. 2017;360(1):201‐205. 27754930 10.1124/jpet.116.237503 PMC5193071

[7] Franke M , Bieber M , Kraft P , Weber A , Stoll G , Schuhmann MK . The NLRP3 inflammasome drives inflammation in ischemia/reperfusion injury after transient middle cerebral artery occlusion in mice. Brain Behav Immun. 2021;92:223‐233. 33307174 10.1016/j.bbi.2020.12.009

[8] Li M , Ransohoff RM . The roles of chemokine CXCL12 in embryonic and brain tumor angiogenesis. Semin Cancer Biol. 2009;19(2):111‐115. 19038344 10.1016/j.semcancer.2008.11.001

[9] Karin N . The multiple faces of CXCL12 (SDF‐1alpha) in the regulation of immunity during health and disease. J Leukoc Biol. 2010;88(3):463‐473. 20501749 10.1189/jlb.0909602

[10] Kim H . Will the stroma‐derived factor‐1α (CXCL12)/CXCR4 pathway become a major concern for advanced colorectal cancer. J Korean Soc Coloproctol. 2012;28(1):3‐4. 22413073 10.3393/jksc.2012.28.1.3 PMC3296939

[11] Luo L , Zang G , Liu B , et al. Bioengineering CXCR4‐overexpressing cell membrane functionalized ROS‐responsive nanotherapeutics for targeting cerebral ischemia‐reperfusion injury. Theranostics. 2021;11(16):8043‐8056. 34335979 10.7150/thno.60785 PMC8315061

[12] Balabanian K , Lagane B , Infantino S , et al. The chemokine SDF‐1/CXCL12 binds to and signals through the orphan receptor RDC1 in T lymphocytes. J Biol Chem. 2005;280(42):35760‐35766. 16107333 10.1074/jbc.M508234200

[13] Kobayashi K , Sato K , Kida T , et al. Stromal cell‐derived factor‐1/C‐X‐C chemokine receptor type 4 axis promotes endothelial cell barrier integrity via phosphoinositide 3‐kinase and Rac1 activation. Arterioscler Thromb Vasc Biol. 2014;34(8):1716‐1722. 24925969 10.1161/ATVBAHA.114.303890

[14] Li X , Zhu M , Penfold ME , et al. Activation of CXCR7 limits atherosclerosis and improves hyperlipidemia by increasing cholesterol uptake in adipose tissue. Circulation. 2014;129(11):1244‐1253. 24374972 10.1161/CIRCULATIONAHA.113.006840

[15] Hao H , Hu S , Chen H , et al. Loss of endothelial CXCR7 impairs vascular homeostasis and cardiac remodeling after myocardial infarction: implications for cardiovascular drug discovery. Circulation. 2017;135(13):1253‐1264. 28154007 10.1161/CIRCULATIONAHA.116.023027

[16] Schönemeier B , Schulz S , Hoellt V , Stumm R . Enhanced expression of the CXCl12/SDF‐1 chemokine receptor CXCR7 after cerebral ischemia in the rat brain. J Neuroimmunol. 2008;198(1‐2):39‐45. 18513805 10.1016/j.jneuroim.2008.04.010

[17] Zhang Y , Zhang H , Lin S , Chen X , Jin K . SDF‐1/CXCR7 chemokine signaling is induced in the peri‐infarct regions in patients with ischemic stroke. Aging Dis. 2018;9(2):287‐295. 29896417 10.14336/AD.2017.1112 PMC5963349

[18] Ehrlich AT , Semache M , Couvineau P . Ackr3‐venus knock‐in mouse lights up brain vasculature. Mol Brain. 2021;14(1):151. 34583741 10.1186/s13041-021-00862-y PMC8477500

[19] Wang Y , Fu W , Zhang S , et al. CXCR‐7 receptor promotes SDF‐1α‐induced migration of bone marrow mesenchymal stem cells in the transient cerebral ischemia/reperfusion rat hippocampus. Brain Res. 2014;1575:78‐86. 24924806 10.1016/j.brainres.2014.05.035

[20] Huang X , Wan M , Yang Q , Ding X , Zhou Z . The stromal cell‐derived factor‐1 α (SDF‐1α)/cysteine‐X‐cysteine chemokine receptor 4 (CXCR4) axis: a possible prognostic indicator of acute ischemic stroke. J Int Med Res. 2019;47(5):1897‐1907. 30760134 10.1177/0300060519827173 PMC6567759

[21] Rohlfing AK , Kolb K , Sigle M , et al. ACKR3 regulates platelet activation and ischemia‐reperfusion tissue injury. Nat Commun. 2022;13(1):1823. 35383158 10.1038/s41467-022-29341-1 PMC8983782

[22] Chai Z , Gong J , Zheng P , Zheng J . Inhibition of miR‐19a‐3p decreases cerebral ischemia/reperfusion injury by targeting IGFBP3 in vivo and in vitro. Biol Res. 2020;53(1):17. 32312329 10.1186/s40659-020-00280-9 PMC7171820

[23] Suofu Y , Wang X , He Y , et al. Mir‐155 knockout protects against ischemia/reperfusion‐induced brain injury and hemorrhagic transformation. Neuroreport. 2020;31(3):235‐239. 31876686 10.1097/WNR.0000000000001382

[24] Aitbaev KA , Murkamilov IT , Fomin VV , Murkamilova JA , Yusupov FA . MicroRNA in ischemic stroke. Zh Nevrol Psikhiatr Im S S Korsakova. 2018;118(3.Vyp.2):48‐56. 10.17116/jnevro20181183248-56 29798981

[25] Xia T , O'Hara A , Araujo I , Barreto J , Harrington WJ . EBV microRNAs in primary lymphomas and targeting of CXCL‐11 by ebv‐mir‐BHRF1‐3. Cancer Res. 2008;68(5):1436‐1442. 18316607 10.1158/0008-5472.CAN-07-5126 PMC2855641

[26] Perry MM , Williams AE , Tsitsiou E , Larner‐Svensson HM , Lindsay MA . Divergent intracellular pathways regulate interleukin‐1β‐induced miR‐146a and miR‐146b expression and chemokine release in human alveolar epithelial cells. FEBS Lett. 2009;583(20):3349‐3355. 19786024 10.1016/j.febslet.2009.09.038

[27] Zhao X , Tang Y , Qu B , Cui H , Shen N . MicroRNA125a contributes to elevated inflammatory chemokine RANTES via targeting KLF13 in systemic lupus erythematosus. Arthritis Rheum. 2010;62(11):3425‐3435. 20589685 10.1002/art.27632

[28] Klein S , Abraham M , Bulvik B , et al. CXCR4 promotes neuroblastoma growth and therapeutic resistance through miR‐15a/16‐1 mediated ERK and BCL2/cyclin D1 pathways. Cancer Res. 2018;78(6):1471‐1483. 29259008 10.1158/0008-5472.CAN-17-0454

[29] Yu X , Wang D , Wang X , et al. CXCL12/CXCR4 promotes inflammation‐driven colorectal cancer progression through activation of RhoA signaling by sponging miR‐133a‐3p. J Exp Clin Cancer Res. 2019;38(1):32. 30678736 10.1186/s13046-018-1014-x PMC6346552

[30] Wang X , Fang Y , Zhou Y , Guo X , Hong Y . SDF‐1a/microRNA‐134 axis regulates nonfunctioning pituitary neuroendocrine tumor growth via targeting VEGFA. Front Endocrinol. 2020;11:566761. 10.3389/fendo.2020.566761 PMC7756115 33362712

[31] Chiang T , Messing RO , Chou WH . Mouse model of middle cerebral artery occlusion. J Vis Exp. 2011;48(48):2761. 10.3791/2761 PMC3197421 21372780

[32] Gerhartl A , Pracser N , Vladetic A , Hendrikx S , Neuhaus W . The pivotal role of micro‐environmental cells in a human blood‐brain barrier in vitro model of cerebral ischemia: functional and transcriptomic analysis. Fluids Barriers CNS. 2020;17(1):19. 32138745 10.1186/s12987-020-00179-3 PMC7059670

[33] Nasoni MG , Carloni S , Canonico B , et al. Melatonin reshapes the mitochondrial network and promotes intercellular mitochondrial transfer via tunneling nanotubes after ischemic‐like injury in hippocampal HT22 cells. J Pineal Res. 2021;71(1):e12747. 34085316 10.1111/jpi.12747 PMC8365755

[34] Mao L , Li P , Zhu W , et al. Regulatory T cells ameliorate tissue plasminogen activator‐induced brain haemorrhage after stroke. Brain. 2017;140(7):1914‐1931. 28535201 10.1093/brain/awx111 PMC6059175

[35] Morris DC , Chopp M , Li Z , Mei L , Zhang ZG . Thymosin beta4 improves functional neurological outcome in a rat model of embolic stroke. Neuroscience. 2010;169(2):674‐682. 20627173 10.1016/j.neuroscience.2010.05.017 PMC2907184

[36] Zhang J , Jiang W , Zuo Z . Pyrrolidine dithiocarbamate attenuates surgery‐induced neuroinflammation and cognitive dysfunction possibly via inhibition of nuclear factor κB. Neuroscience. 2014;261:1‐10. 24365462 10.1016/j.neuroscience.2013.12.034 PMC3950371

[37] Amano M , Hamaguchi T , Shohag MH , et al. Kinase‐interacting substrate screening is a novel method to identify kinase substrates. J Cell Biol. 2015;209(6):895‐912. 26101221 10.1083/jcb.201412008 PMC4477863

[38] Rhodes LV , Bratton MR , Zhu Y , et al. Effects of SDF‐1‐CXCR4 signaling on microRNA expression and tumorigenesis in estrogen receptor‐alpha (ER‐α)‐positive breast cancer cells. Exp Cell Res. 2011;317(18):2573‐2581. 21906588 10.1016/j.yexcr.2011.08.016 PMC3334320

[39] Potter ML , Smith K , Vyavahare S , et al. Characterization of differentially expressed miRNAs by CXCL12/SDF‐1 in human bone marrow stromal cells. Biomol Concepts. 2021;12(1):132‐143. 34648701 10.1515/bmc-2021-0015

[40] Lipfert J , Odemis V , Wagner DC , Boltze J , Engele J . CXCR4 and CXCR7 form a functional receptor unit for SDF‐1/CXCL12 in primary rodent microglia. Neuropathol Appl Neurobiol. 2013;39(6):667‐680. 23289420 10.1111/nan.12015

[41] Banisadr G , Podojil JR , Miller SD , Miller RJ . Pattern of CXCR7 gene expression in mouse bain under normal and inflammatory conditions. J Neuroimmune Pharmacol. 2016;11(1):26‐35. 25997895 10.1007/s11481-015-9616-y PMC4831709

[42] Cheng X , Wang H , Zhang X , et al. The role of SDF‐1/CXCR4/CXCR7 in neuronal regeneration after cerebral ischemia. Front Neurosci. 2017;11:590. 29123467 10.3389/fnins.2017.00590 PMC5662889

[43] Yang F , Luo WJ , Sun W , et al. SDF1‐CXCR4 signaling maintains central post‐stroke pain through mediation of glial‐neuronal interactions. Front Mol Neurosci. 2017;10:226. 28785202 10.3389/fnmol.2017.00226 PMC5519565

[44] Liu ZY , Song ZW , Guo SW , et al. CXCL12/CXCR4 signaling contributes to neuropathic pain via central sensitization mechanisms in a rat spinal nerve ligation model. CNS Neurosci Ther. 2019;25(9):922‐936. 30955244 10.1111/cns.13128 PMC6698967

[45] Gong P , Zhang Z , Zou C , et al. Hippo/YAP signaling pathway mitigates blood‐brain barrier disruption after cerebral ischemia/reperfusion injury. Behav Brain Res. 2019;356:8‐17. 30092249 10.1016/j.bbr.2018.08.003 PMC6193462

