# Supplementary material for: Chemokine receptor 7 mediates miRNA‐182 to regulate cerebral ischemia/reperfusion injury in rats
Source: CNS Neurosci Ther. 2022 Dec 15;29(2):712–26. doi: 10.1111/cns.14056 (PMC9873520; doi:10.1111/cns.14056)

Fig. 1. (C)

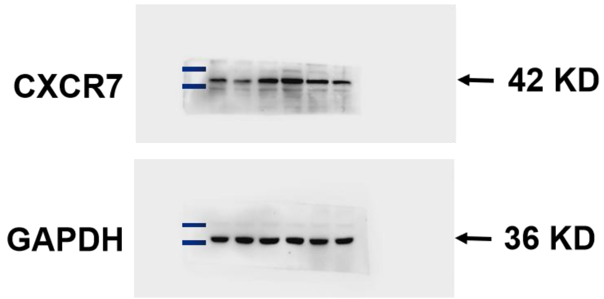

Fig. 1. (E)

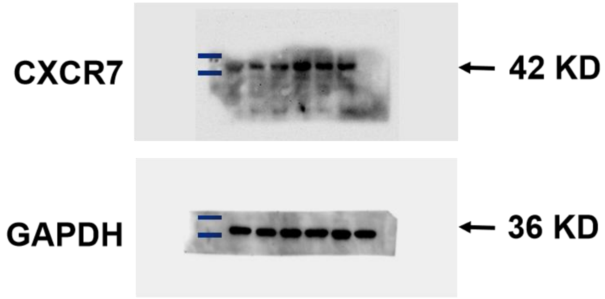

Fig. 3. (E)

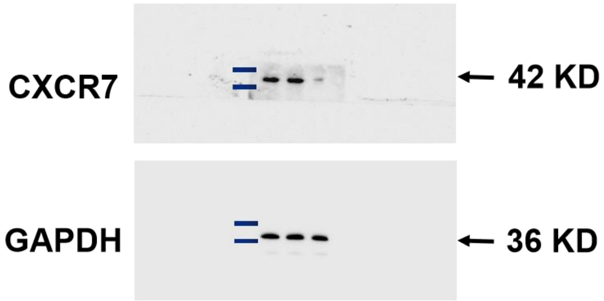

Fig. 5. (D)

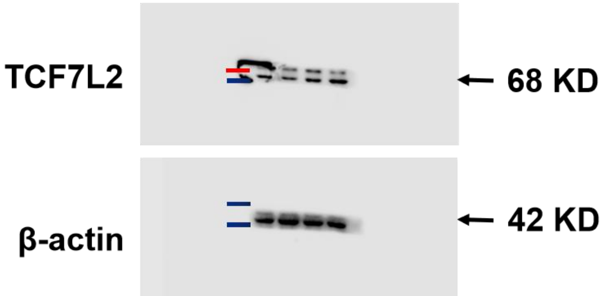

Fig. 5. (F)

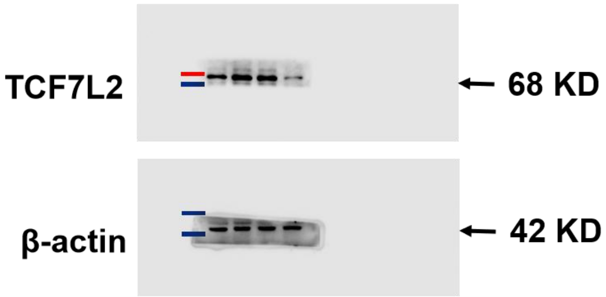

Fig. 6. (B)

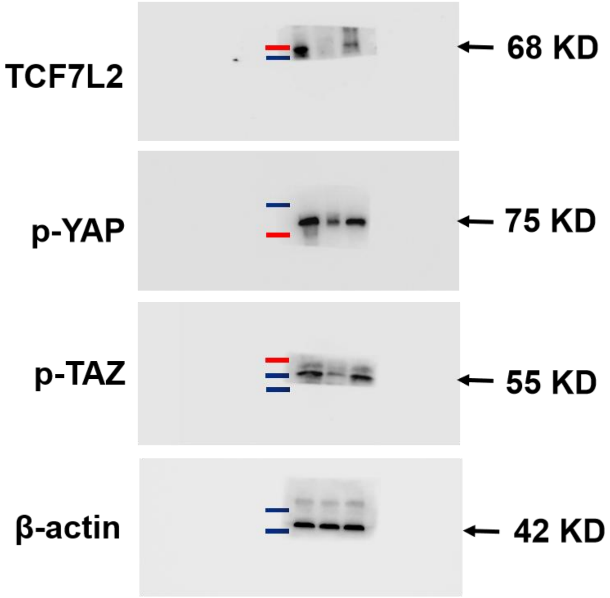

Supplemental Figure S7.(A)

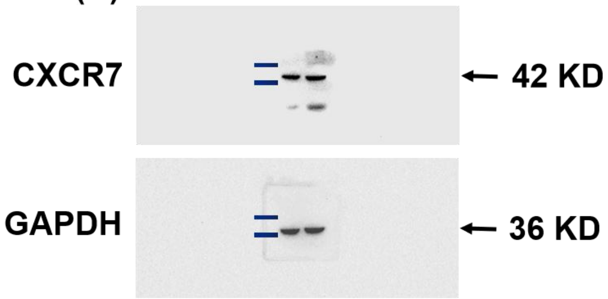

Supplement: Supplementary file 1 — AppendixS1 [file CNS-29-712-s002.pdf]
