# Supplementary material for: Chemokine receptor 7 mediates miRNA‐182 to regulate cerebral ischemia/reperfusion injury in rats
Source: CNS Neurosci Ther. 2022 Dec 15;29(2):712–26. doi: 10.1111/cns.14056 (PMC9873520; doi:10.1111/cns.14056)
Supplement: Supplementary file 2 — AppendixS2 [file CNS-29-712-s001.docx]

**Chemokine receptor 7 mediates miRNA-182 to regulate cerebral ischemia/reperfusion injury in rats**

Supplementary Information

**Supplementary Methods and Materials**

**Inclusion and exclusion criteria**

The participants or their legally authorized representatives provided written informed consent. For individuals in the AIS group, participants were required to meet the following criteria: (1) acute neurological deficit within 6 hours of admission; (2) computed tomographic excluded brain hemorrhage; (3) infarction located in the middle cerebral artery (MCA) region and M1 segment of MCA occlusion as determined by magnetic resonance angiography, digital subtraction angiography (DSA) or computed tomography angiography; (4) receiving thrombolysis therapy or mechanical thrombectomy; (5) DSA confirmed vascular recanalization; Exclusion criteria for the AIS group included the following: (1) Exclusion criteria for the AIS group included the following: undergoing decompressive craniectomy; (2) surgery or trauma history within 3 months; (3) acute myocardial infarction or other acute ischemic events history within the past 3 months; (4) other cancer or related immune diseases history.

Healthy control subjects were recruited from healthy examination subjects. Exclusion criteria include history of head trauma, major diseases, drug abuse.

**Brain tissue excision method**

Brain tissues from the ischemic penumbra in MCAO rats and the corresponding regions in sham rats were harvested for further experiments. As described previously^1,2^, a 2 mm section was cut 5 mm from the anterior tip of the frontal lobe. A longitudinal cut was made from top to bottom ~2 mm from the midline through the ischemic hemisphere of this section. Then, a transverse diagonal cut was made at approximately the “1 o′clock” position to separate the core from the penumbra in the adjacent cortex.

**
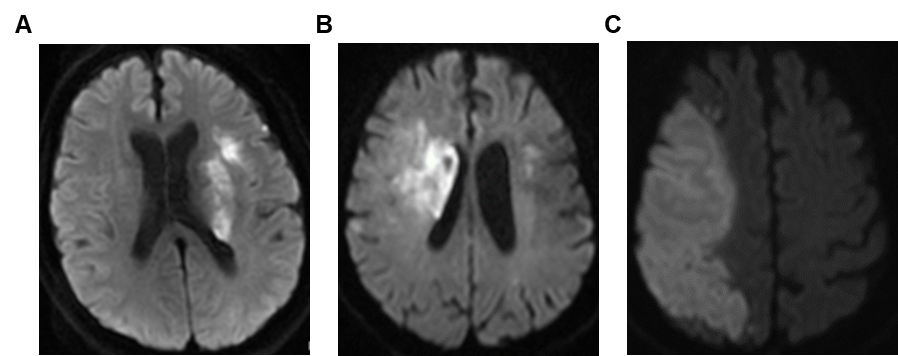
**

**Supplemental Figure S1.** Classification of infarction volume according to the size of the MCA territory by DWI and FLAIR sequence of 3.0 T nuclear magnetic resonance. (A) Small volume, ischemic changes of < one third of the MCA territory. (B) Middle volume, ischemic changes between one third and < two thirds of the MCA territory. (C) Large volume, ischemic changes of two thirds of the MCA territory. MCA, middle cerebral artery.


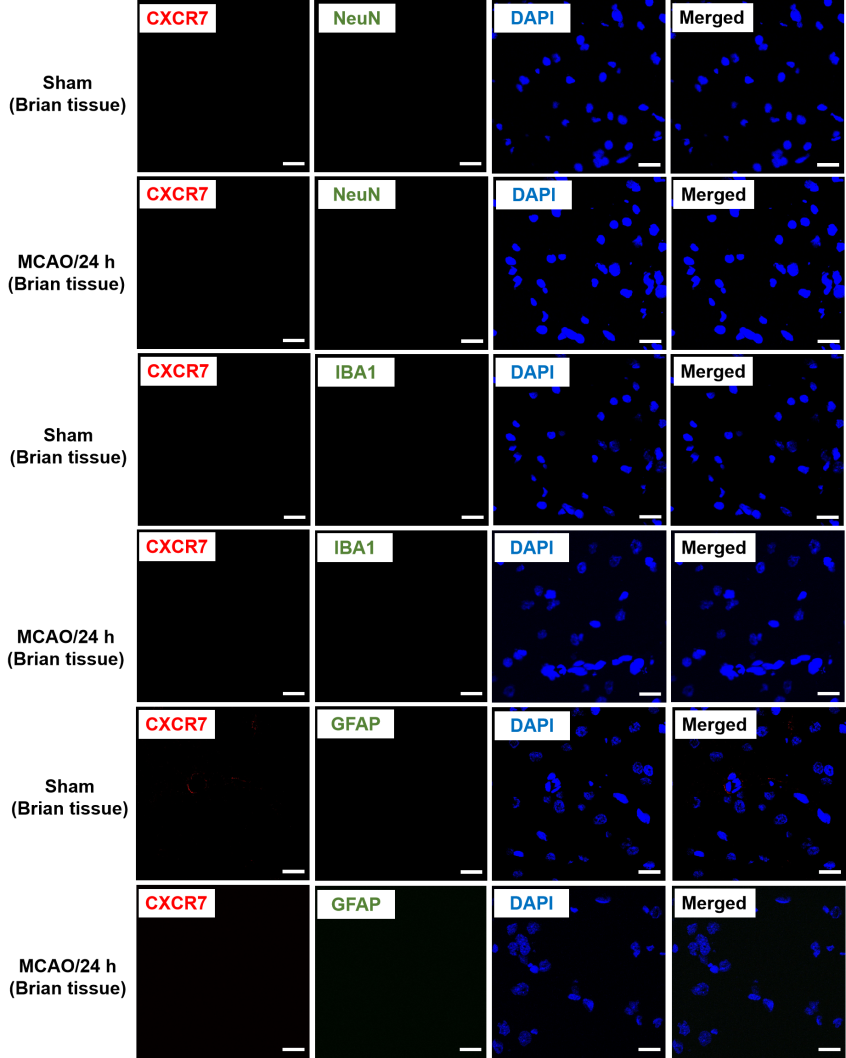


**Supplemental Figure S2.** CXCR7 is expressed in neurons, microglia, and astrocytes. Co-localization of CXCR7 with NeuN, Iba1 and GFAP after sham, MCAO. Representative images of immunofluorescence showed background staining of CXCR7, NeuN, Iba1, and GFAP, including areas in which they overlapped. Scale bar: 20 μm. Brain tissue samples were obtained from the ischemic penumbra area 24 h after MCAO (n = 3).


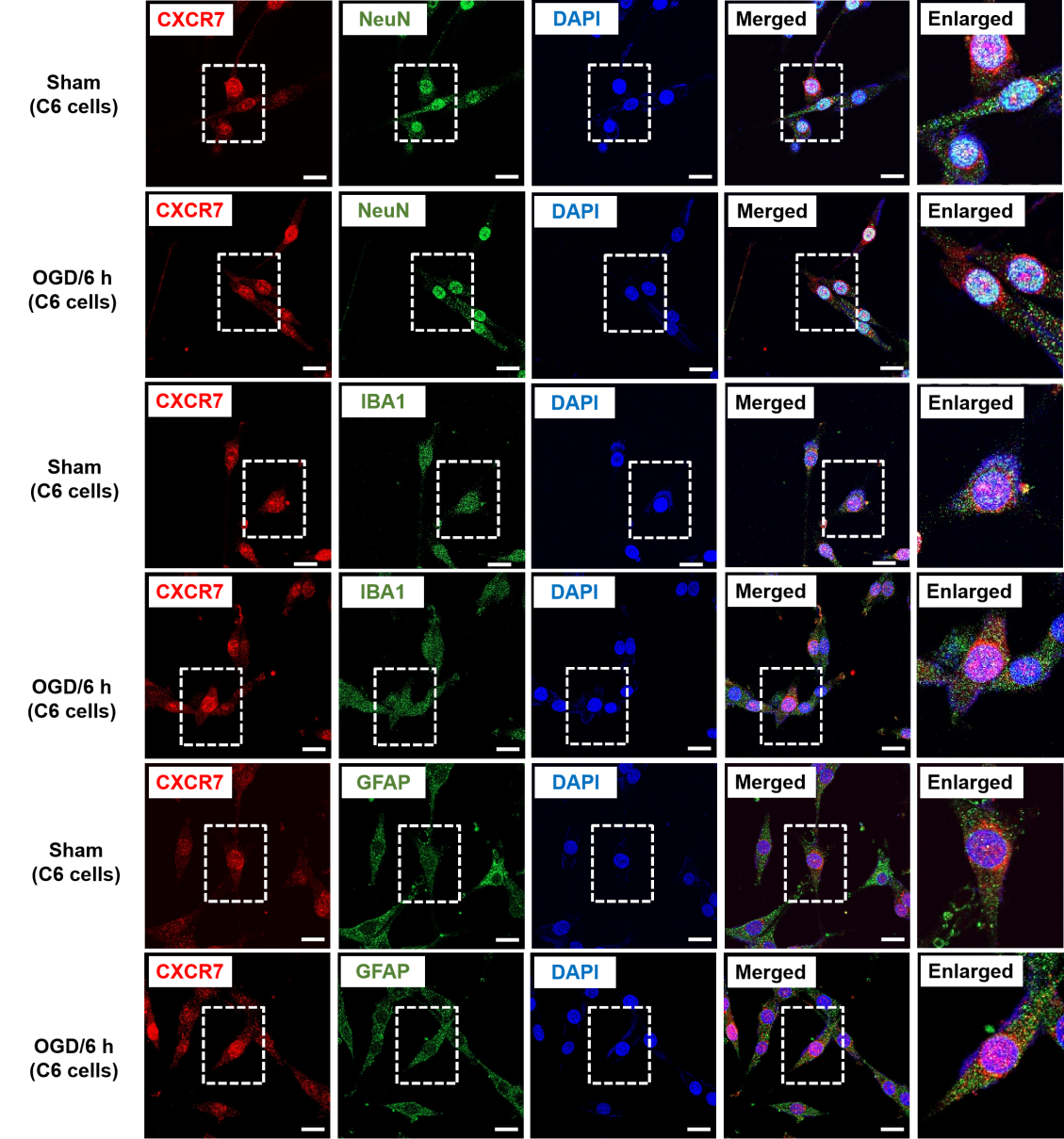


**Supplemental Figure S3.** CXCR7 is expressed in neurons, microglia, and astrocytes. Co-localization of CXCR7 with NeuN, Iba1 and GFAP after sham, OGD. Representative images of immunofluorescence staining showed the expression of CXCR7 (red), NeuN (green), Iba1 (green), and GFAP (green), including areas in which they overlapped, shown in rectangles. Scale bar: 20 μm. C6 cells samples were obtained 6 h after OGD (n = 3).


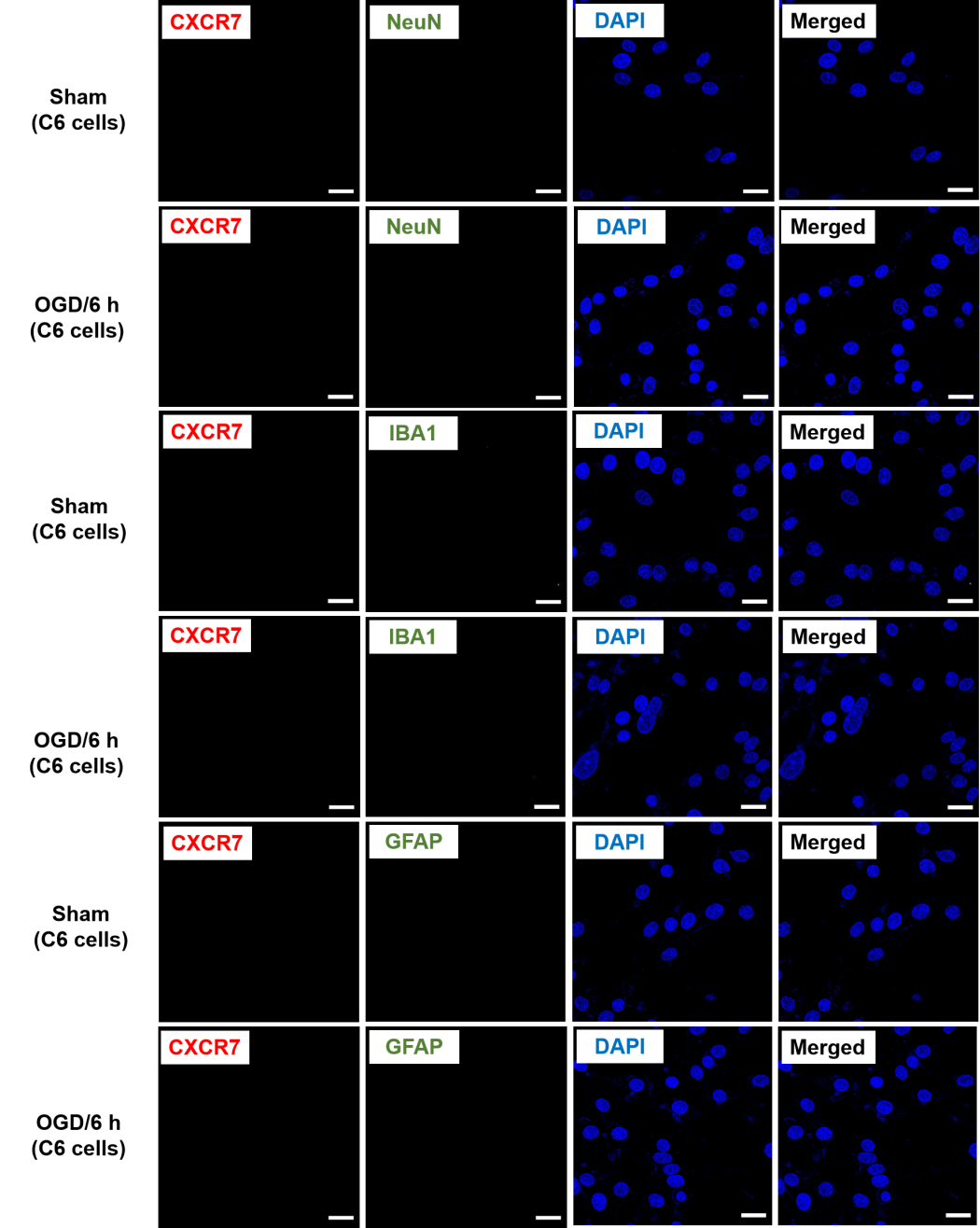


**Supplemental Figure S4.** CXCR7 is expressed in neurons, microglia, and astrocytes. Co-localization of CXCR7 with NeuN, Iba1 and GFAP after sham, OGD. Representative images of immunofluorescence showed the background staining of CXCR7, NeuN, Iba1, and GFAP, including areas in which they overlapped. Scale bar: 20 μm. C6 cells samples were obtained 6 h after OGD (n = 3).


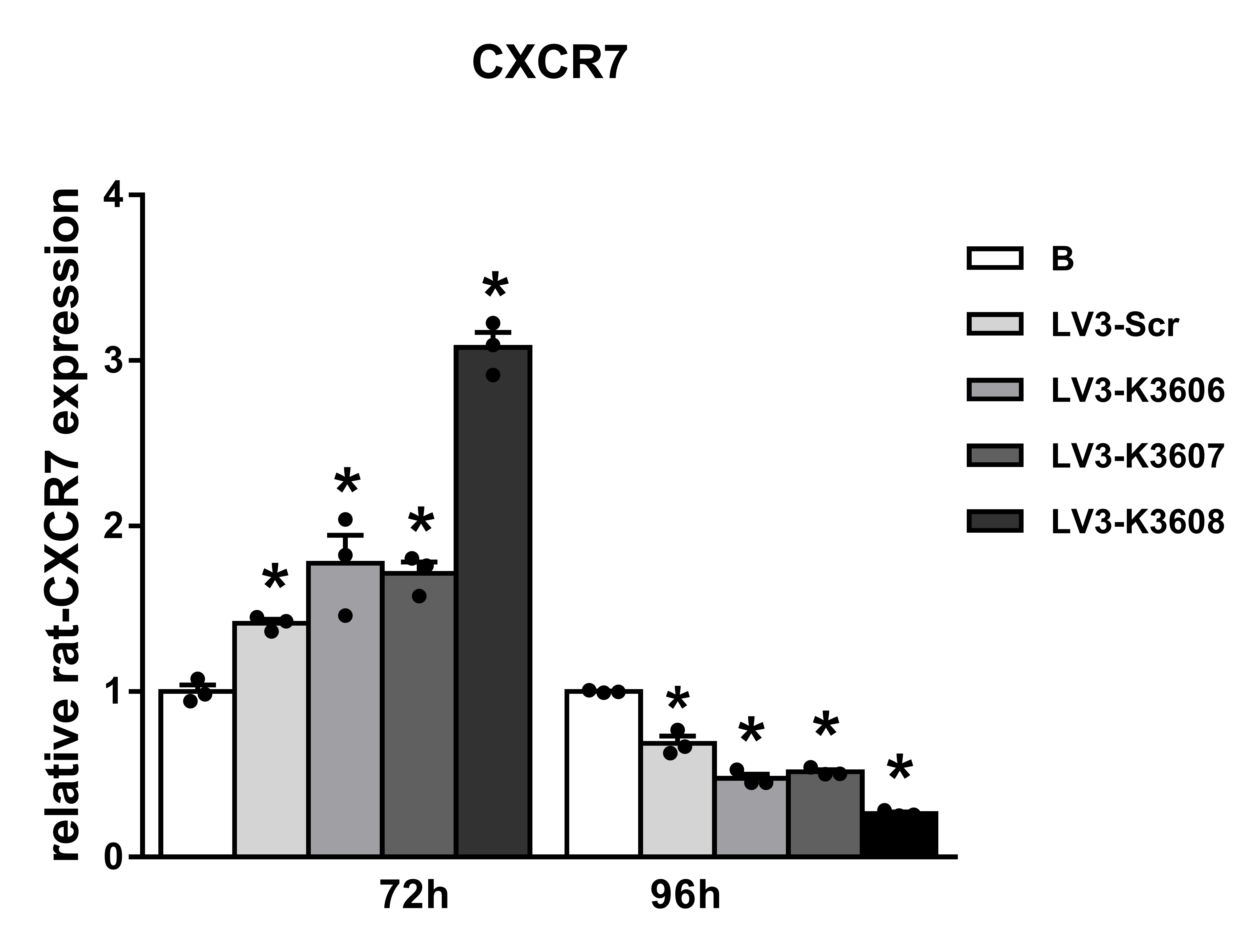


**Supplemental Figure S5.** Rat-CXCR7 gene expression level was calibrated with rat-ACTB as the internal reference. B represents blank control group, LV3-Scr represents shRNA-Scr group, LV3-K3606 represents shRNA-CXCR7 sequence 1, LV3-K3607 represents shRNA-CXCR7 sequence 2, and LV3-K3608 represents shRNA-CXCR7 sequence 3. There were 3 replicates for each gene in each sample, and the precision met the requirements of further analysis. Data were expressed as mean ±SEM, **P* < 0.05 *vs.* the sham group, n = 3.


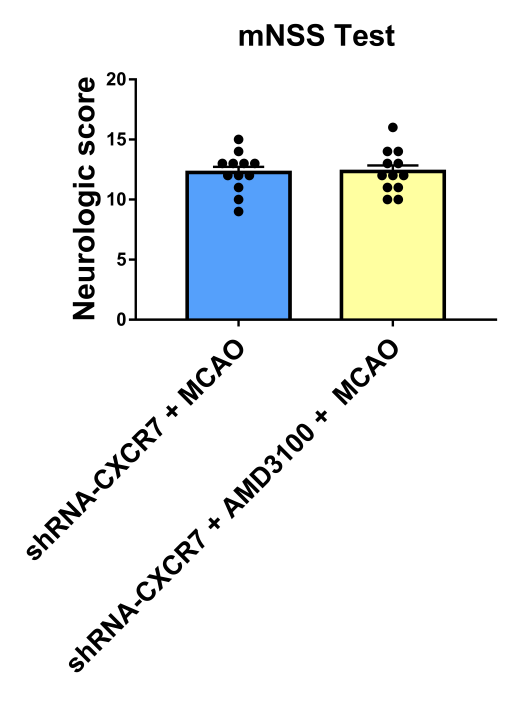


**Supplemental Figure S6.** mNSS test in the shRNA-CXCR7 + MCAO and shRNA-CXCR7 + AMD3100 + MCAO groups (n = 12). The data are expressed as the means ± SEM. The data were analyzed by T-test. *p* > 0.05 *vs.* the shRNA-CXCR7 + MCAO group. mNSS, the modified neurologic severity score.


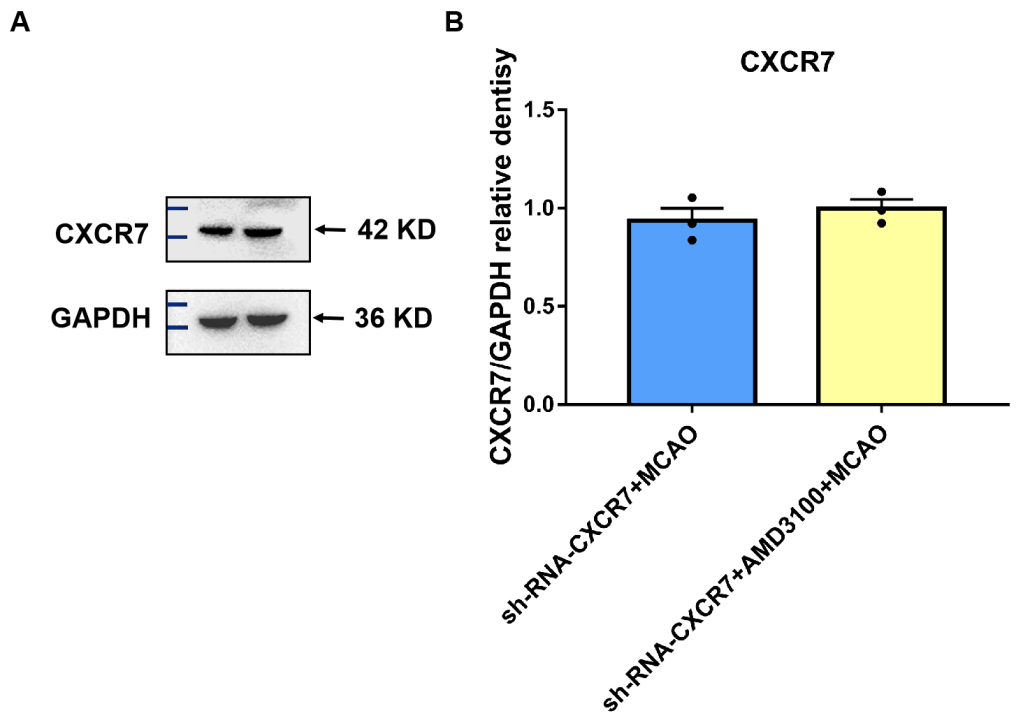


**Supplemental Figure S7.** (A) Representative western blot bands showing CXCR7 expression in the shRNA-CXCR7 + MCAO and shRNA-CXCR7 + AMD3100 + MCAO groups (n = 3). (B) Densitometric quantification of CXCR7/β-actin. The data are expressed as the means ± SEM. The data were analyzed by T-test. *p* > 0.05 *vs.* the shRNA-CXCR7 + MCAO group.


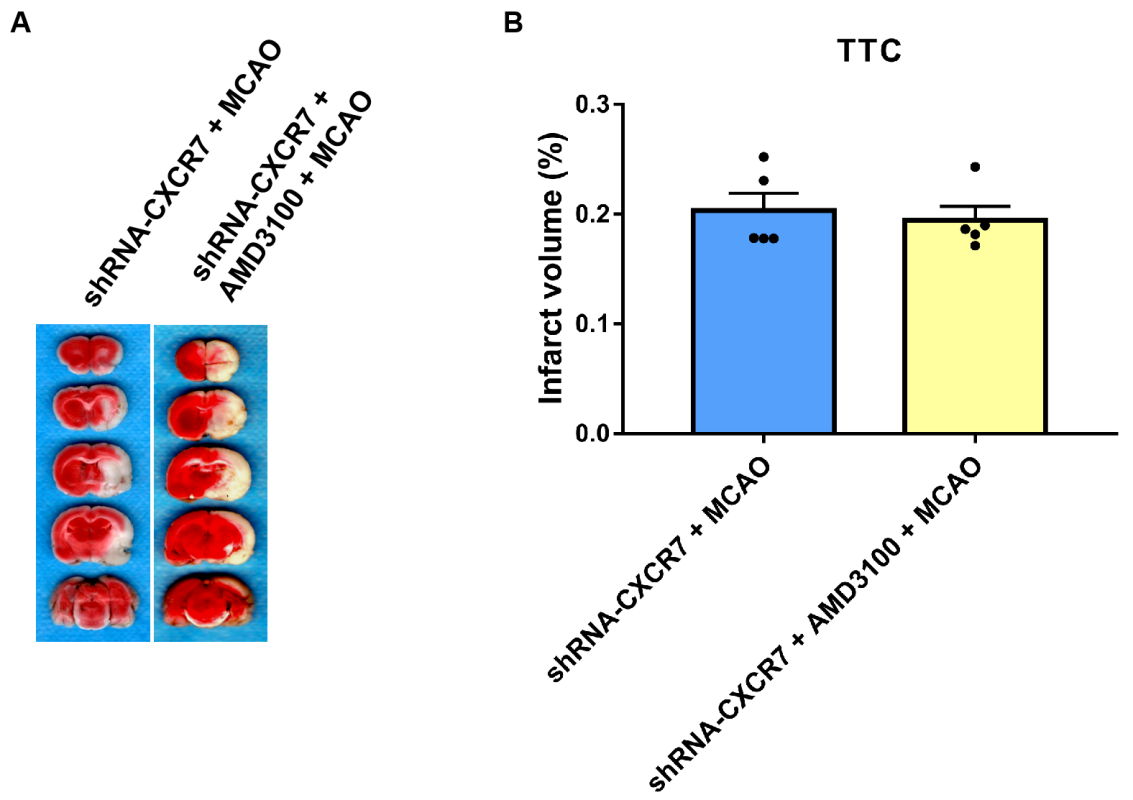


**Supplemental Figure S8.** (A)TTC-stained brain sections and (B) quantitative data illustrating the increased infarct volume in the shRNA-CXCR7 + MCAO and shRNA-CXCR7 + amd3100 + MCAO groups (n = 5). The data are expressed as the means ± SEM. The data were analyzed by T-test. *p* > 0.05 *vs.* the shRNA-CXCR7 + MCAO group. TTC, 2,3,5-triphenyltetrazolium chloride.


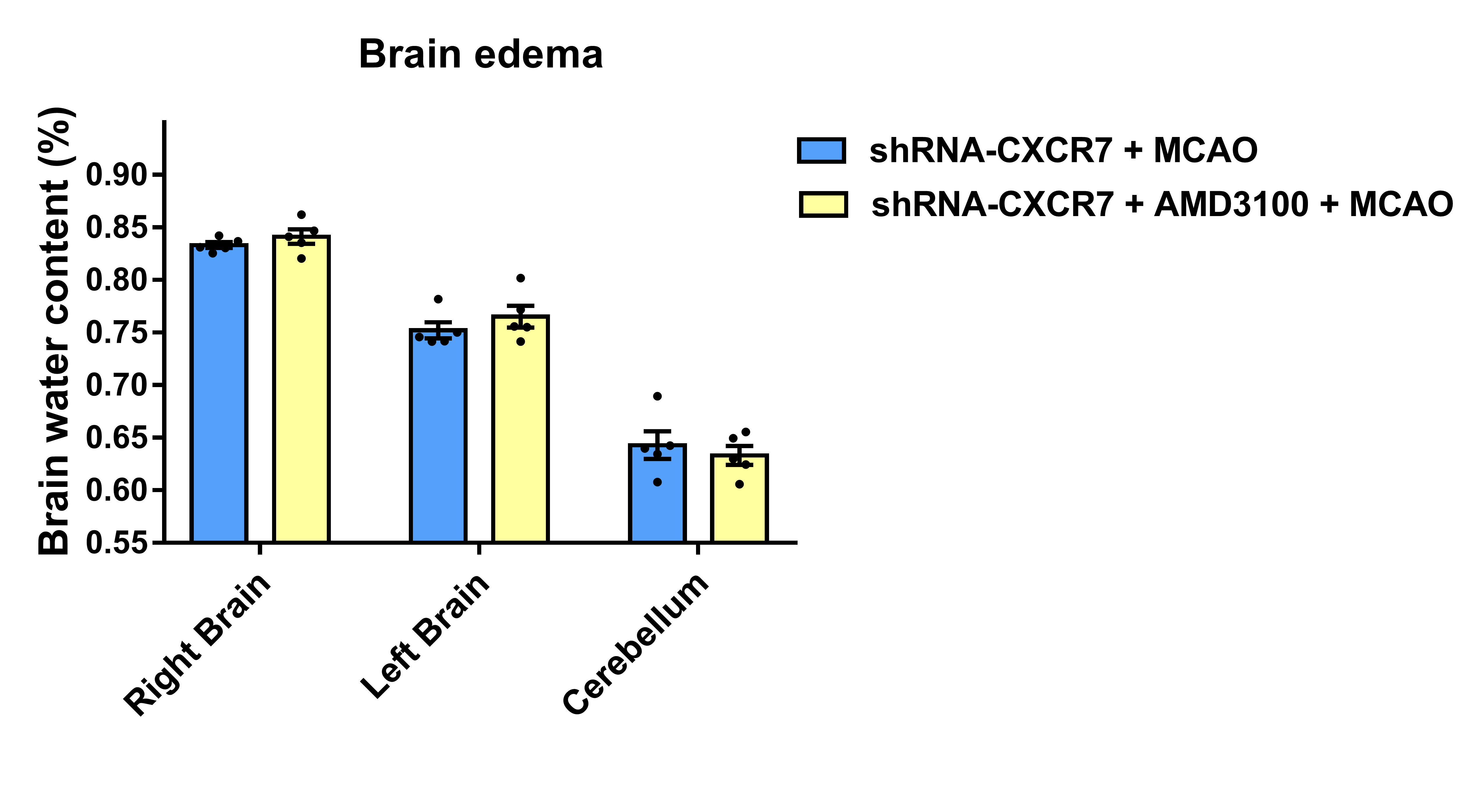


**Supplemental Figure S9.** Brain edema 24 h after surgery in the sham, MCAO, vehicle + MCAO, and shRNA-CXCR7 + MCAO groups (n = 5). The brain was divided into three parts: the right brain, left brain, and cerebellum. The data are expressed as the means ± SEM. The data were analyzed by T-test. *p* > 0.05 *vs.* the shRNA-CXCR7 + MCAO group.


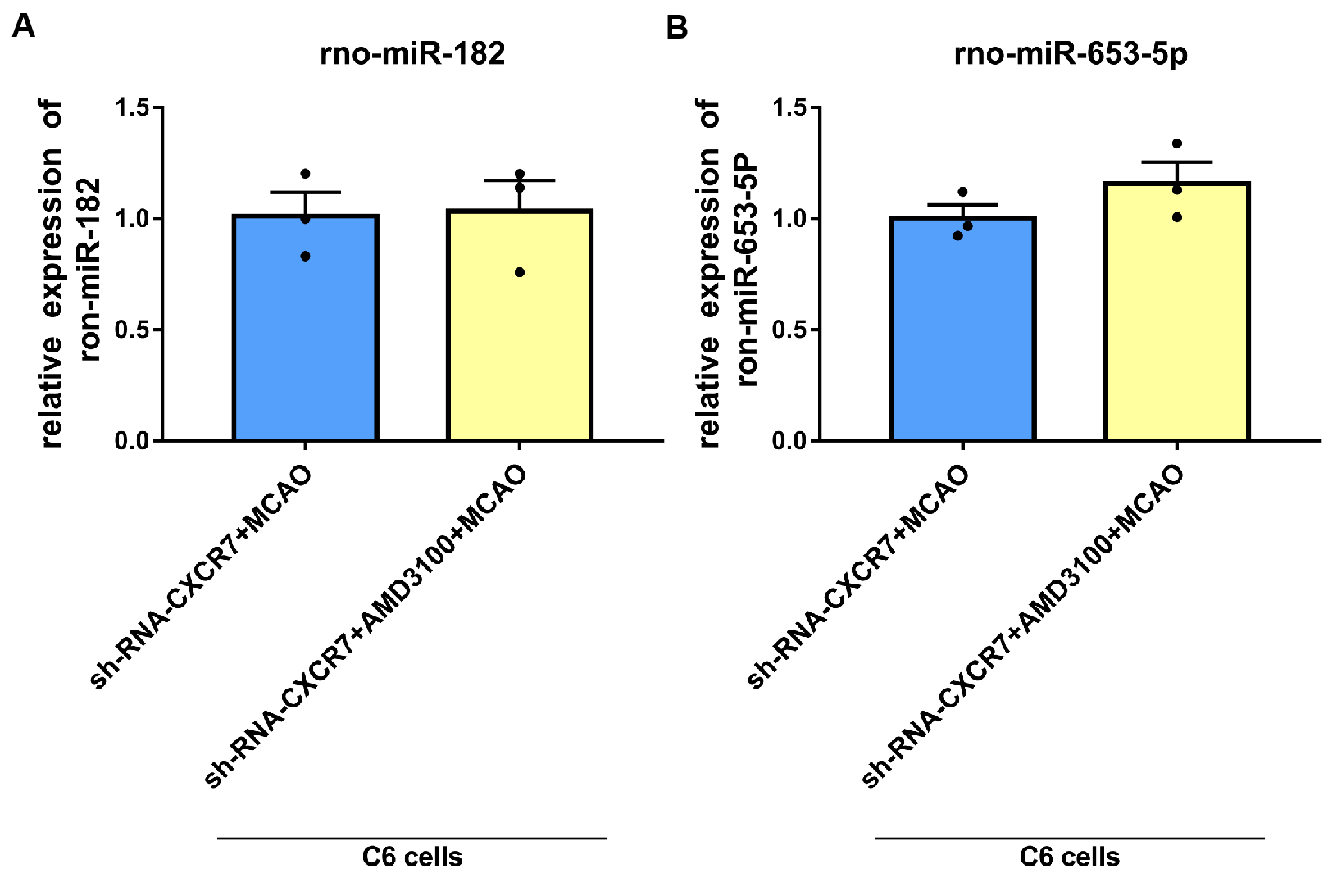


**Supplemental Figure S10.** (A) The levels of miR-182 decreased by qRT-PCR in brain tissues after MCAO with knocking down CXCR7 and CXCR4 treatment (n = 3) compared with those in the shRNA-CXCR7 + MCAO group (n = 3). (B) The level of miR-653-5P detected by qRT-PCR in brain tissues after MCAO with knocking down CXCR7 and CXCR4 treatment (n = 3) compared with those in the shRNA-CXCR7 + MCAO group (n = 3). All data are expressed as the means ± SEM. *p* > 0.05 *vs.* the shRNA-CXCR7 + MCAO group.


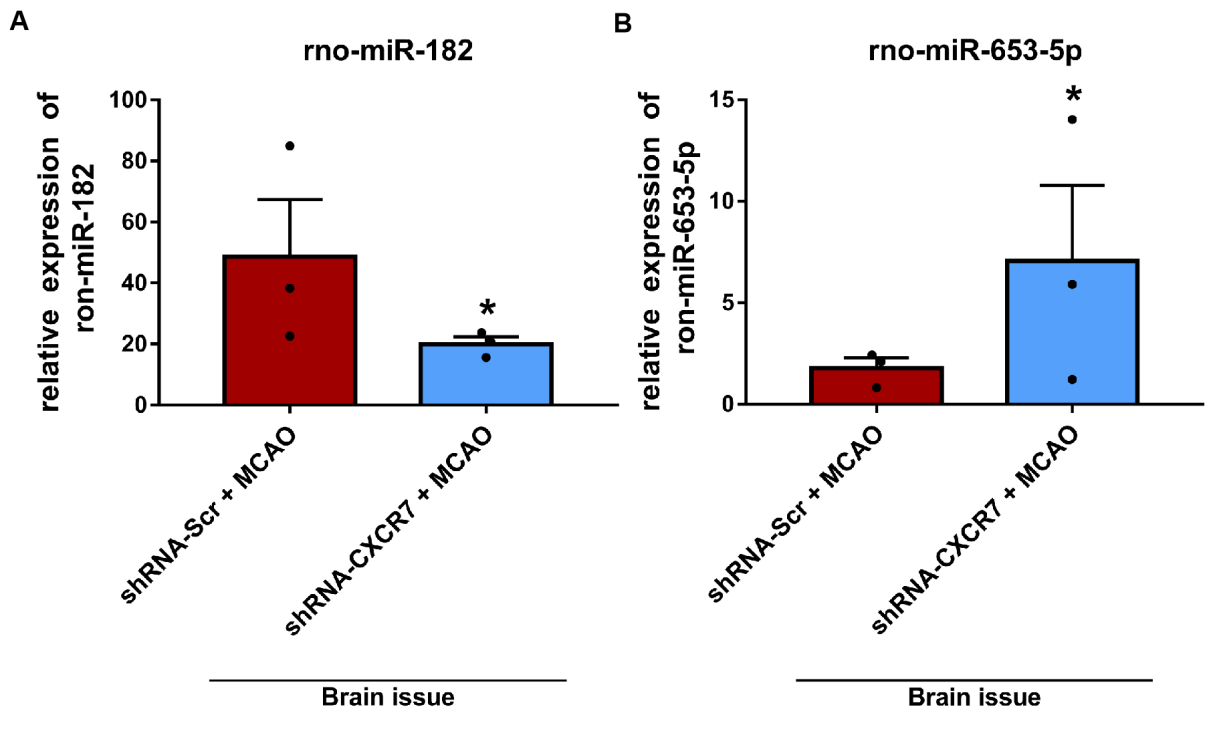


**Supplemental Figure S11.** Validation of miRNAs microarray data. (A) The level of miR-182 was detected by miRNA microarray. (B) The level of miR-653-5P was detected by miRNA microarray.


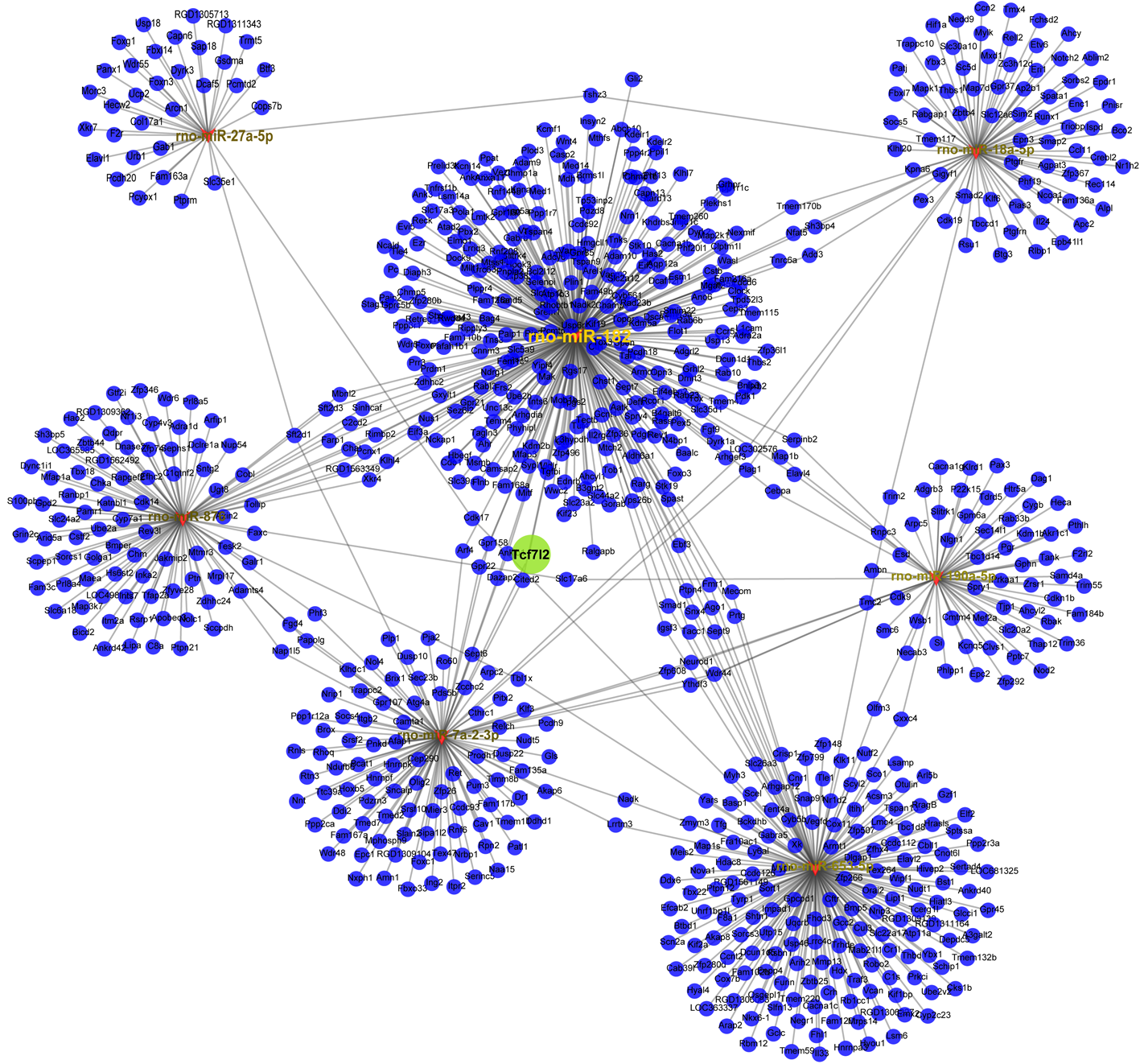


**Supplemental Figure S12.** Information network for miRNA prediction of downstream target genes. Presumed connections between miRNAs and CXCR7-associated target genes according to the miRNA degree, and miR-182 was the most significant.


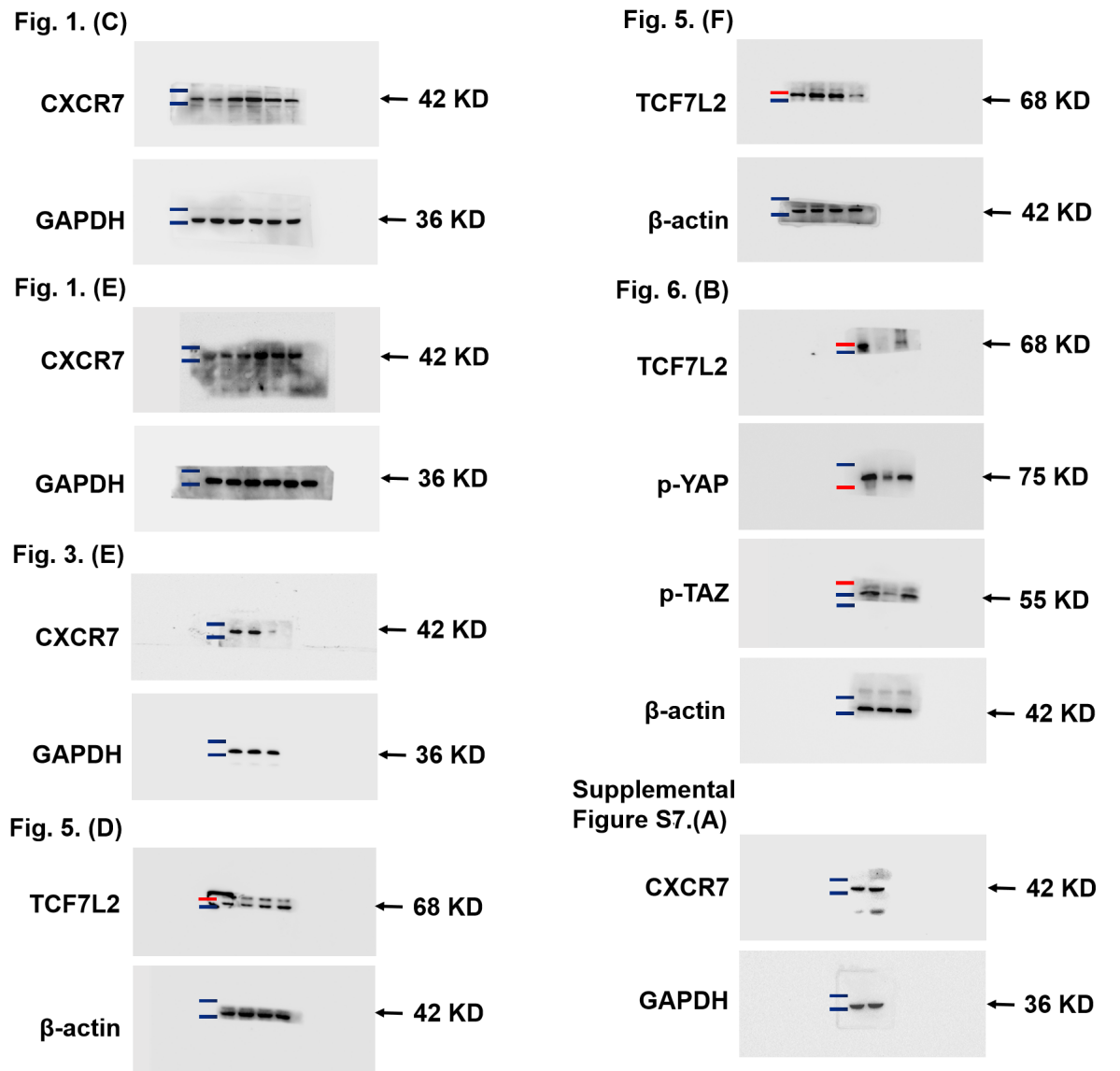


**Supplemental Figure S13.** The original gel/blot images of each figure.

**Supplemental Table S1.** Zea-longa score

| **Tests** | **Points** |
| --- | --- |
| No neurological deficit  Failure to extend contralateral forepaw  Spin longitudinally  Falling to the contralateral side  Unable to walk spontaneously | 0  1  2  3  4 |
| Maximum points | 4 |

Rats with a score between 2 and 3 were considered successful models and then included in subsequent researches.

**Supplemental** **Table S2.** Modified neurologic severity scores (mNSS)

| **Tests** | **Points** |
| --- | --- |
| **Motor tests**  Raising the rat by the tail  Flexion of forelimb  Flexion of hindlimb  Head moving more than 10° (vertical axis)  Placing the rat on the floor  Inablity to walk straight  Circling toward the paretic side  Falling down to the paretic side | 1  1  1  1  1  1 |
| **Sensory tests**  Visual and tactile placing  Proprioceptive test (deep sensory) | 1  1 |
| **Beam balance tests**  Grasps side of beam  Hugs the beam and one limb falls down from the beam  Hugs the beam and two limbs fall down from the beam, or spins on beam (<60 seconds)  Attempts to balance on the beam but falls off (<40 seconds)  Attempts to balance on the beam but falls off (<20 seconds)  Falls off: no attempt to balance or hang on to the beam (<20 seconds) | 1  2  3  4  5  6 |
| **Reflexes (blunt or sharp stimulation) absent of:**  Pinna reflex (a head shake when touching the auditory meatus)  Corneal reflex (an eye blink when lightly touching the cornea with cotton)  Startle reflex (a motor response to a brief loud paper noise)  Seizures, myoclonus, myodystony | 1  1  1  1 |
| Maximum points | 18 |

One point is awarded for the inability to perform the tasks or for the lack of a tested reflex; 1 to 6 indicates mild injury; 7 to 12 indicates moderate injury; 13 to 18 indicates severe injury.

**Supplemental Table S3.** Baseline characteristics of the 27 included patients

| **Characteristic** | **ALL**  N = 27 | **AIS group**  N = 17 | **Control group**  N = 10 | **Statistical**  **test** | ***P*-value** |
| --- | --- | --- | --- | --- | --- |
| **Age, y** (SD) | 65.0(13.9) | 70.8(8.9) | 55.2(15.8) | 2.864 | 0.014 |
| **Sex, male**, n (%) | 11(40.7) | 8(47.1) | 3(30.0) |  | 0.448 |
| **Medical History,** n (%) |  |  |  |  |  |
| Hypertension | 13(48.1) | 11(64.7) | 2(20.0) |  | 0.046 |
| Diabetes **Mellitus** | 6(22.2) | 4(23.5) | 2(20.0) |  | 1.000 |
| Atrial fibrillation | 11(40.7) | 10(58.8) | 1(10.0) |  | 0.018 |
| History of Stroke | 6(22.2) | 4(23.5) | 2(20.0) |  | 1.000 |
| Hyperlipemia | 3(11.1) | 2(11.8) | 1(10.0) |  | 1.000 |

AIS, acute ischemic stroke

**Supplemental Table S4.** The differentially expressed miRNAs in shRNA-CXCR7 + MCAO group compared with shRNA-NC + MCAO group

| **miRNAs** | **Singal value** | | **Fold change** |  | **FDR** |
| --- | --- | --- | --- | --- | --- |
|  | **(mean ± SEM, n = 3)** | | **(shRNA-CXCR7 + MCAO/shRNA + MCAO)** | ***P-*value** | **adjusted** |
|  | **shRNA-Scr + MCAO** | **shRNA-CXCR7 + MCAO** |  |  | ***P-*value** |
| rno-miR-653-5p | 0.780045884 | 2.84639206 | 4.188245983 | 2.70e-02 | 1.00e+00 |
| rno-miR-876 | -0.747959526 | 1.117477901 | 3.643783968 | 2.85e-02 | 1.00e+00 |
| rno-miR-18a-5p | 0.42557602 | 2.060518089 | 3.105750788 | 8.35e-03 | 1.00e+00 |
| rno-miR-7a-2-3p | 3.851107916 | 4.838773364 | 1.982973568 | 1.44e-02 | 1.00e+00 |
| rno-miR-190a-5p | 4.634917228 | 5.369219746 | 1.663592999 | 4.93-02 | 1.00e+00 |
| rno-miR-182 | 5.613481294 | 4.324118909 | 0.40913181 | 1.77e-02 | 1.00e+00 |
| rno-miR-27a | 4.788122865 | 3.967458313 | 0.566181081 | 4.73e-02 | 1.00e+00 |

MCAO: middle cerebral artery occlusion; FDR, false discovery rate.

**References**

[1] Ashwal S, Tone B, Tian HR, Cole DJ, Pearce WJ. Core and penumbral nitric oxide synthase activity during cerebral ischemia and reperfusion. Stroke. 1998. 29(5): 1037-46; discussion 1047.

[2] Moskowitz MA, Lo EH, Iadecola C. The science of stroke: mechanisms in search of treatments. Neuron. 2010. 67(2): 181-98.
